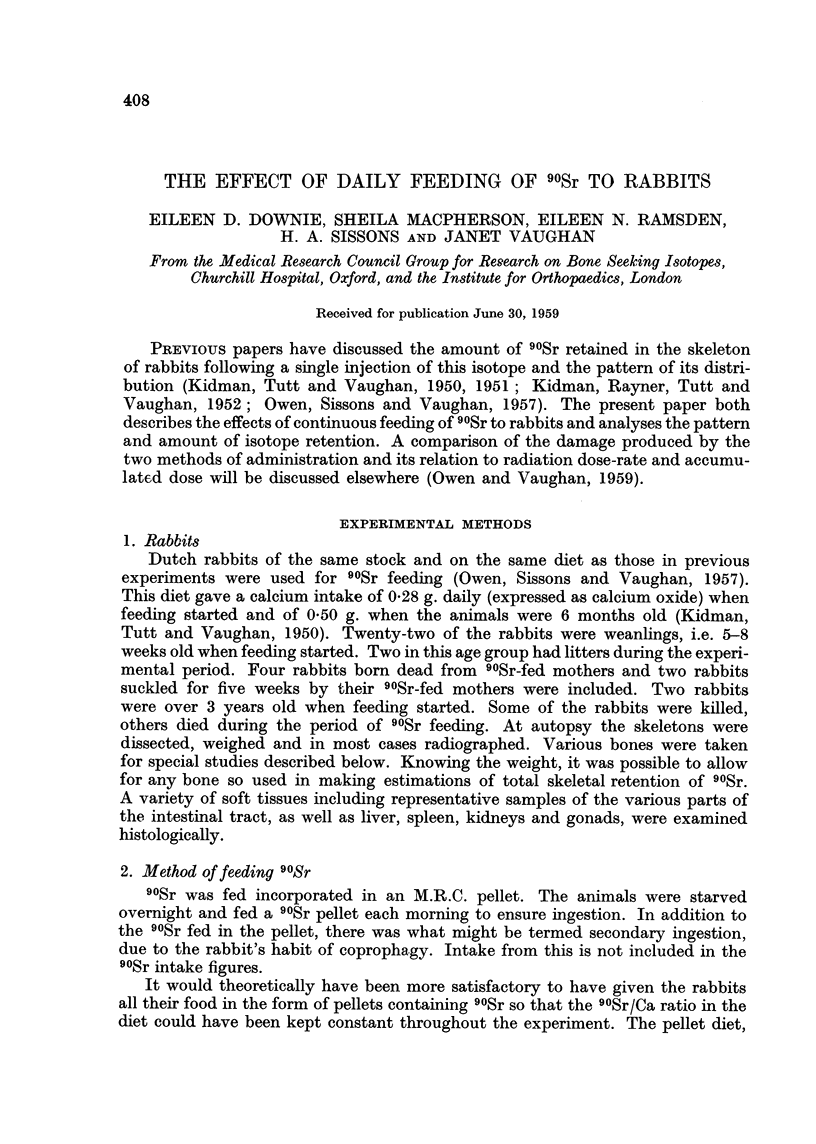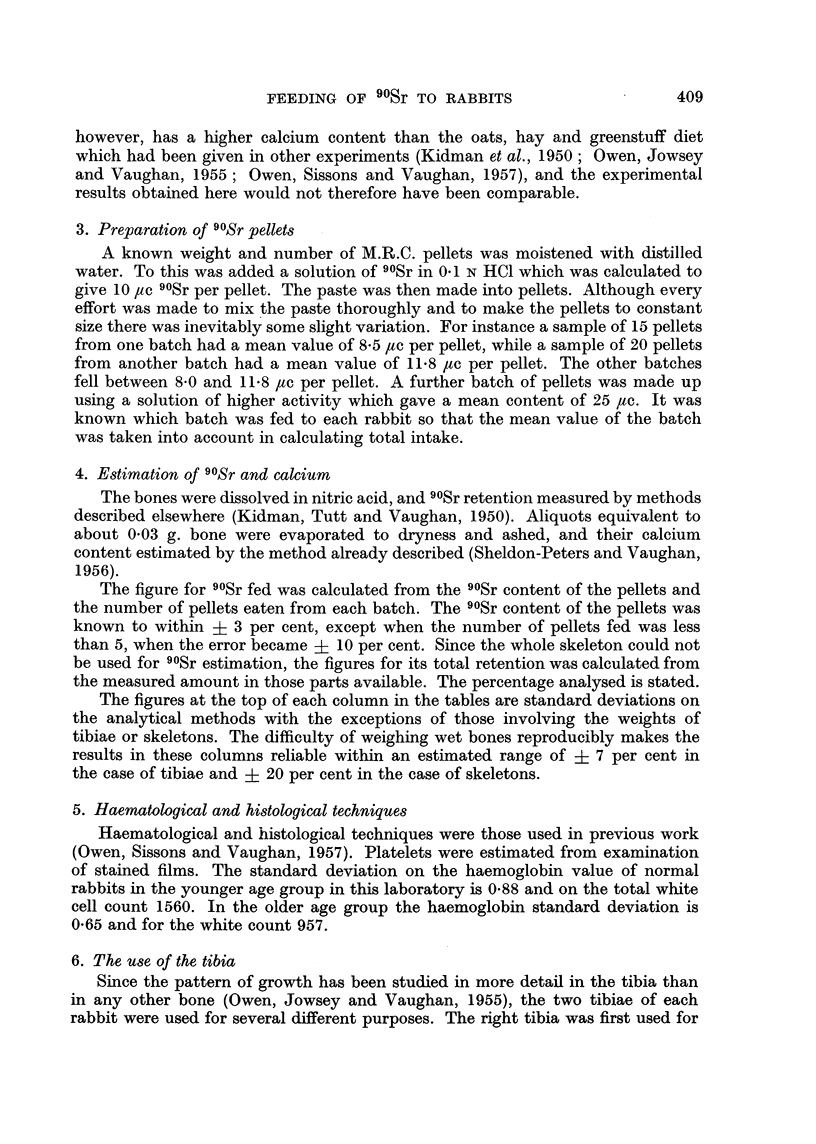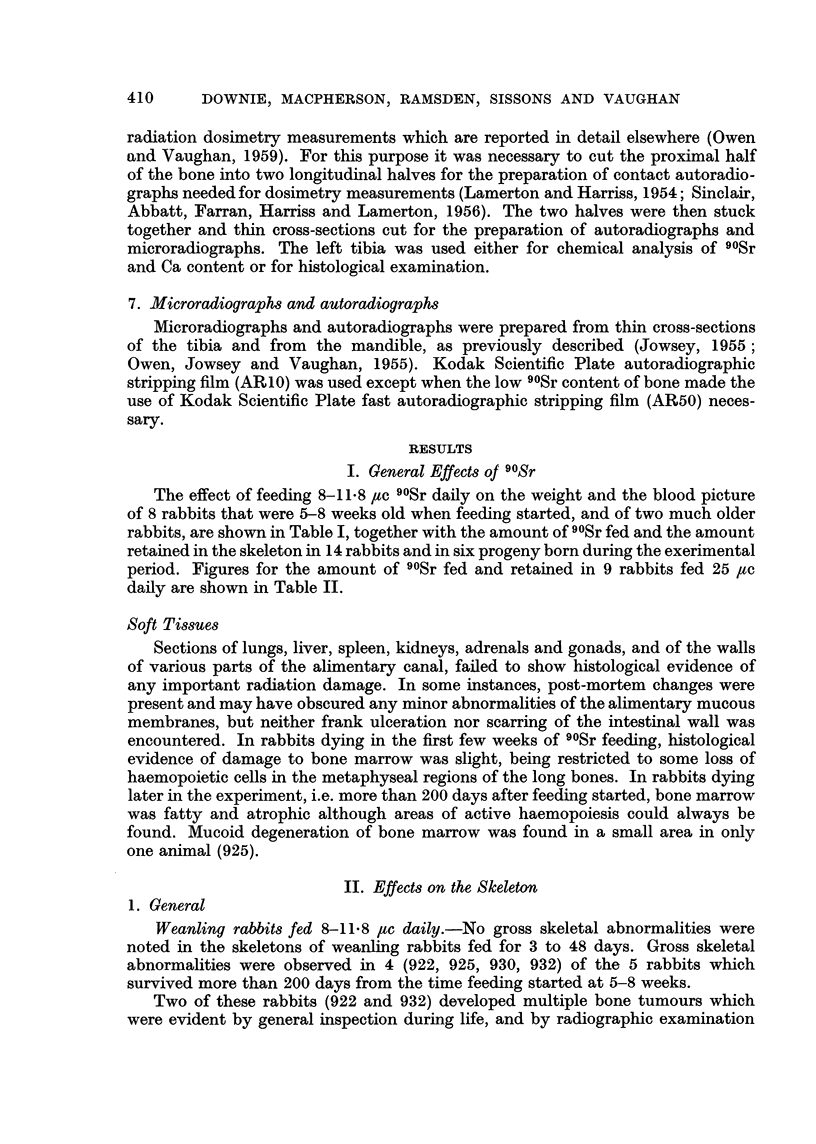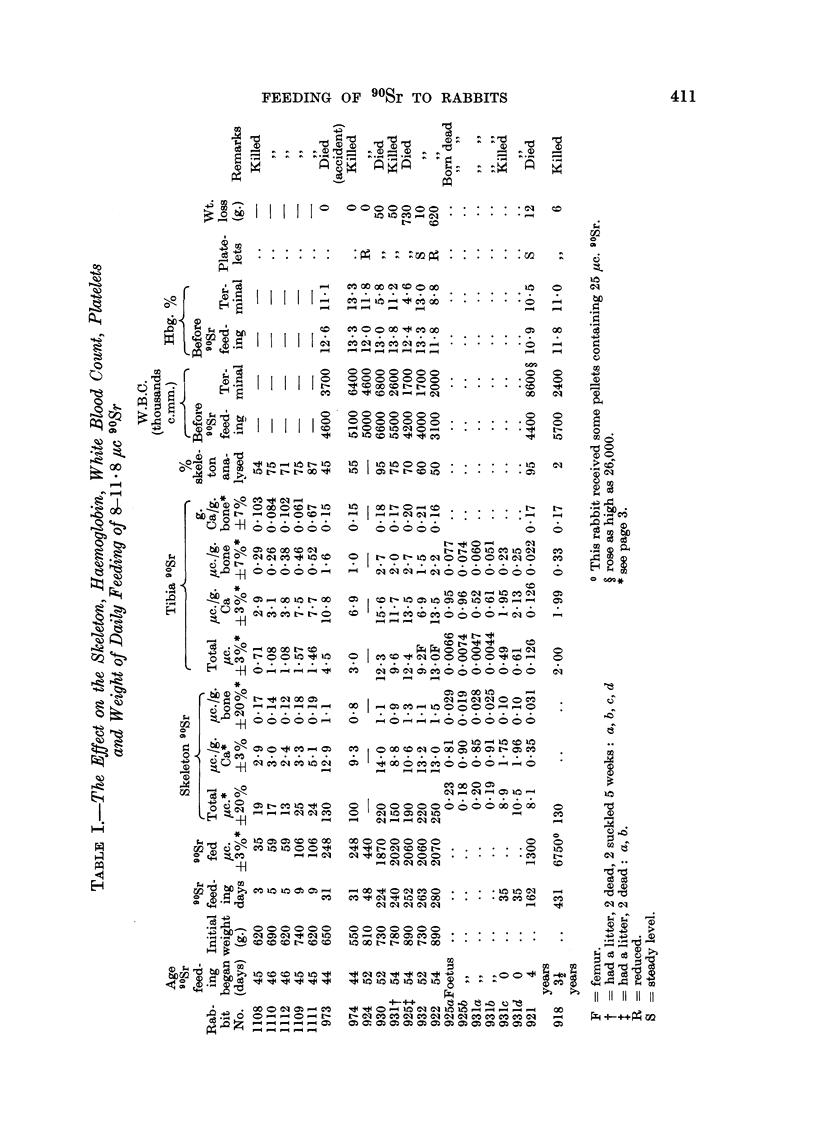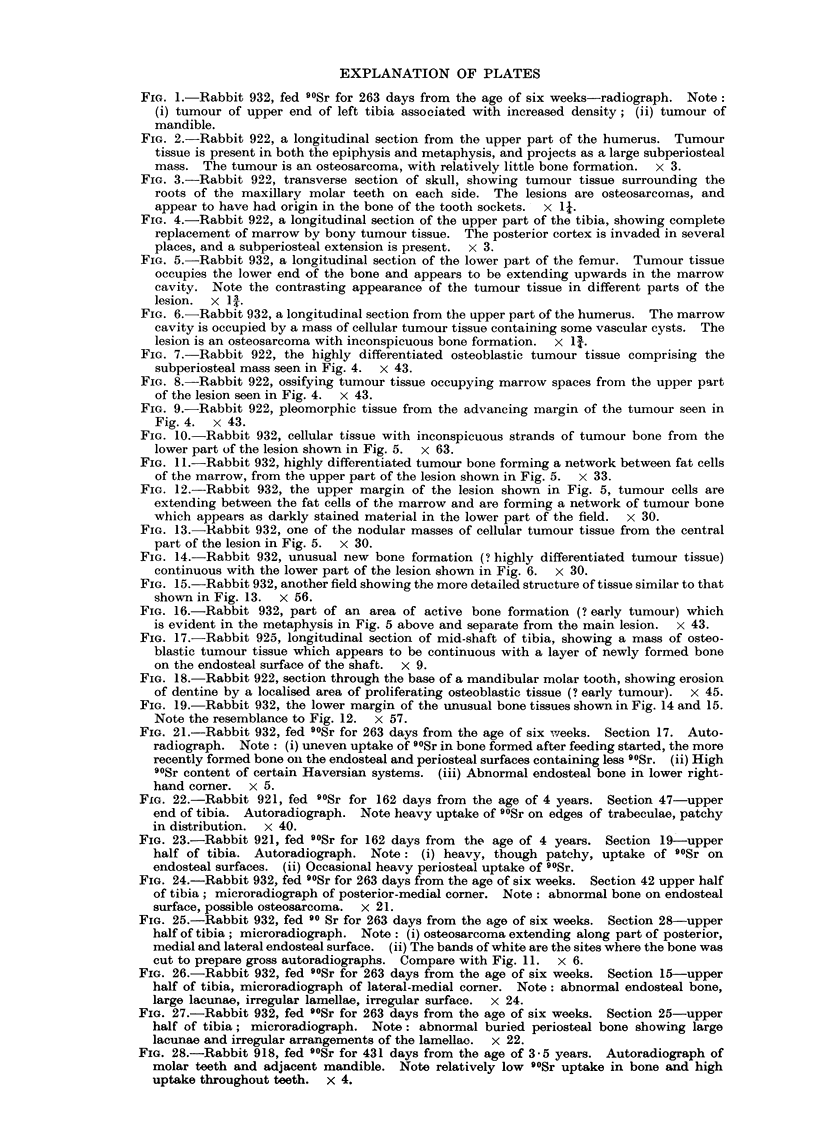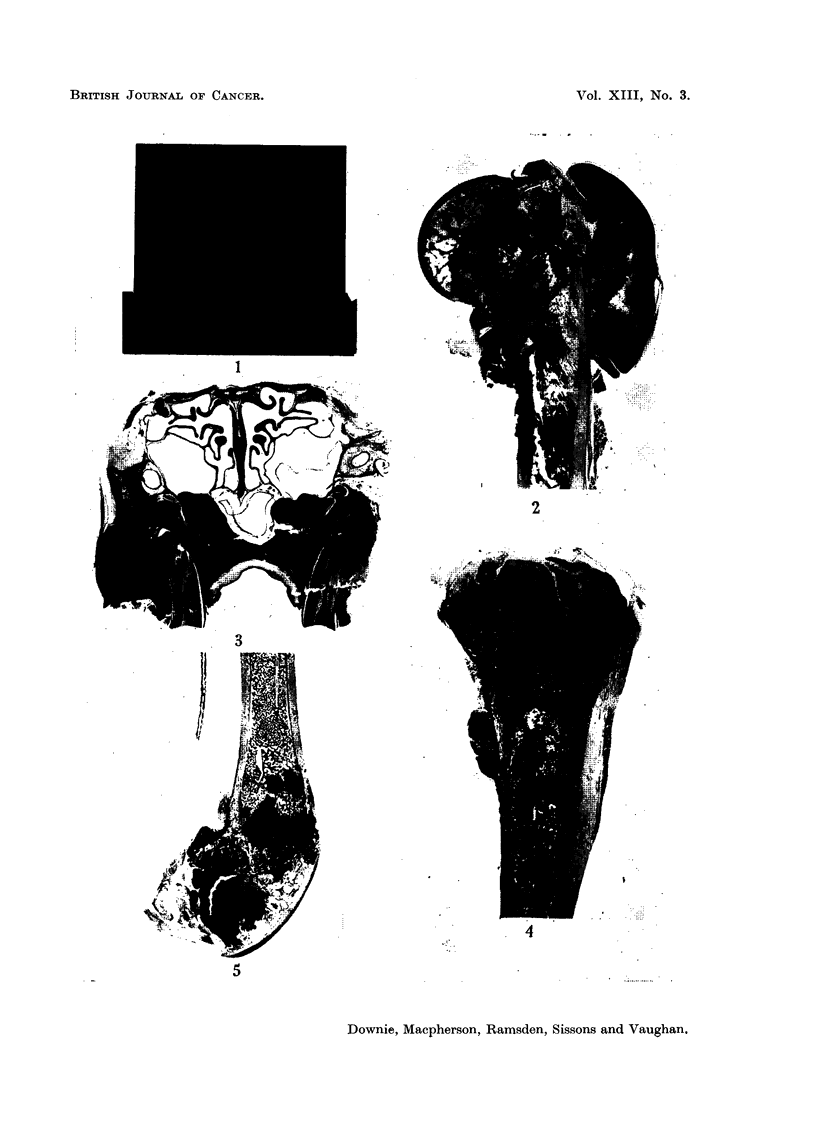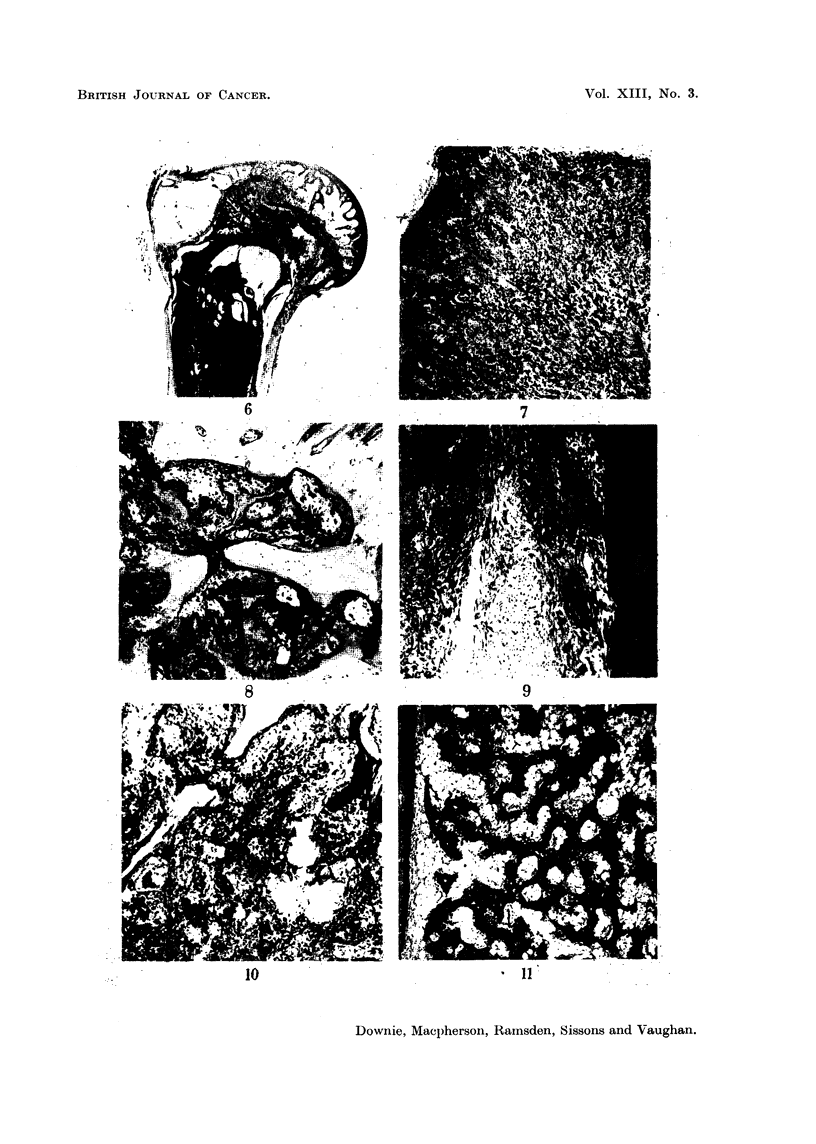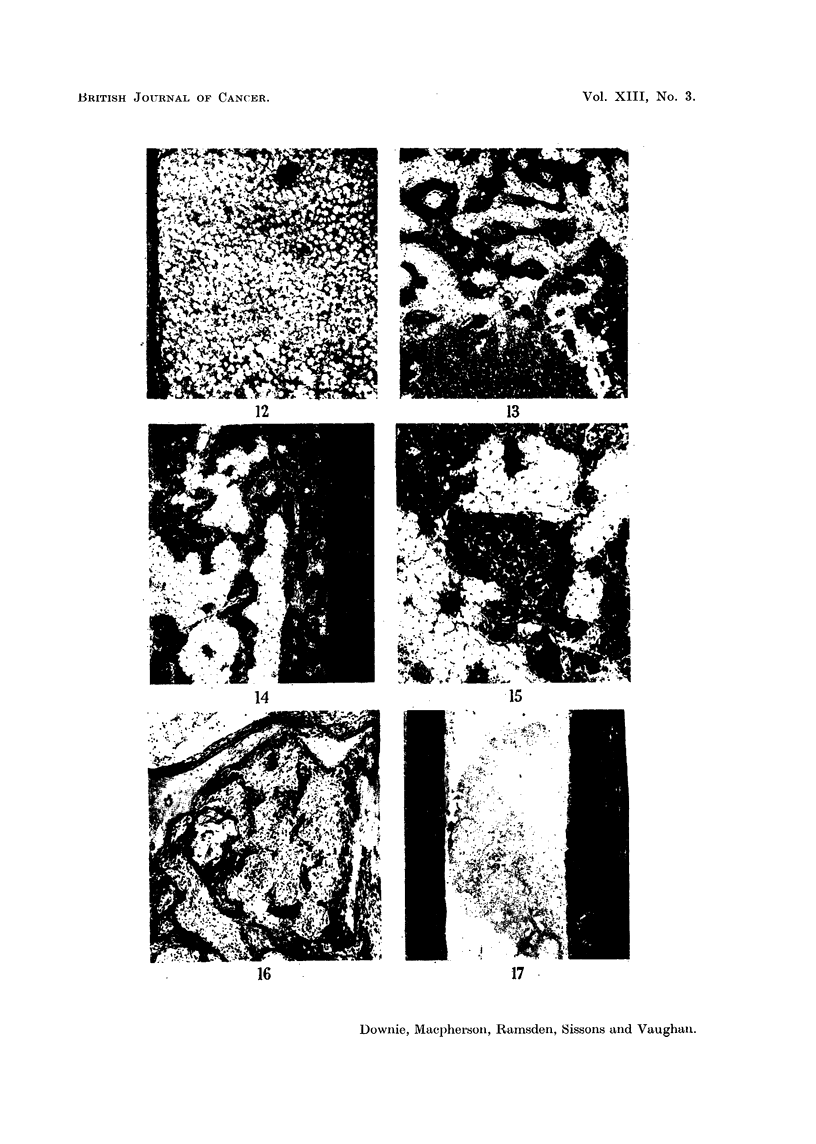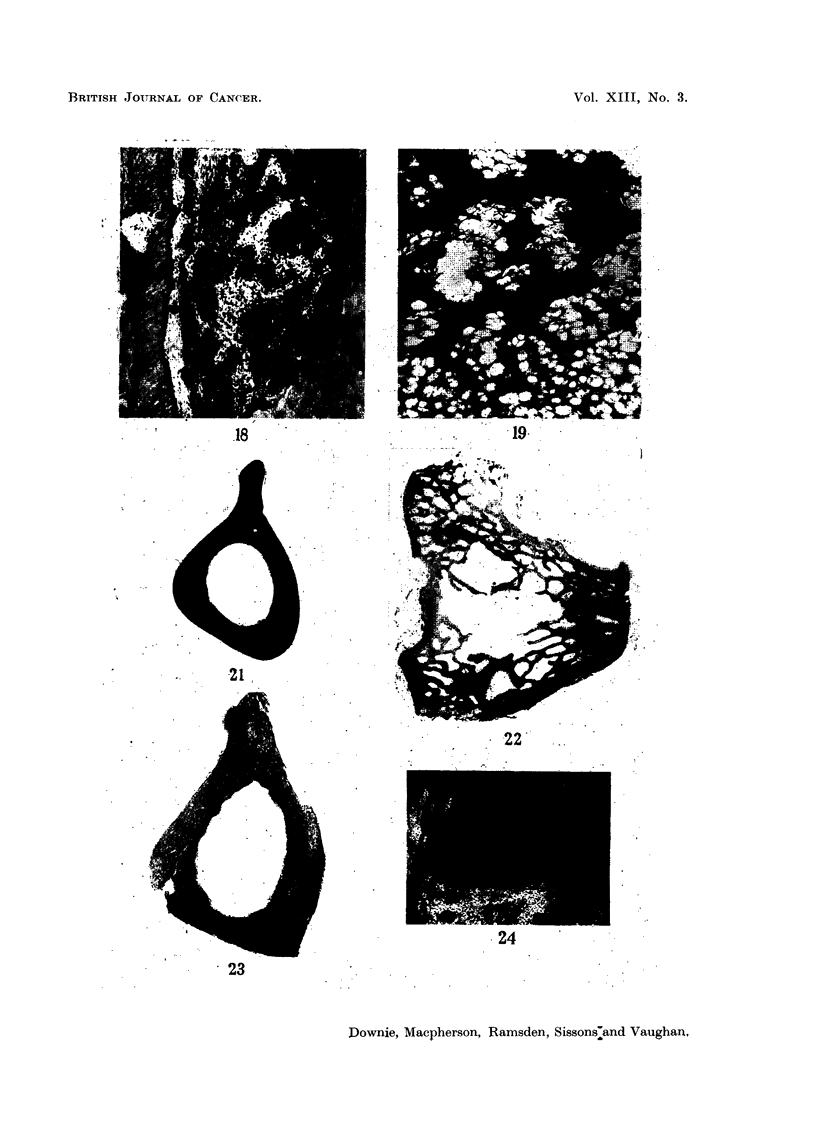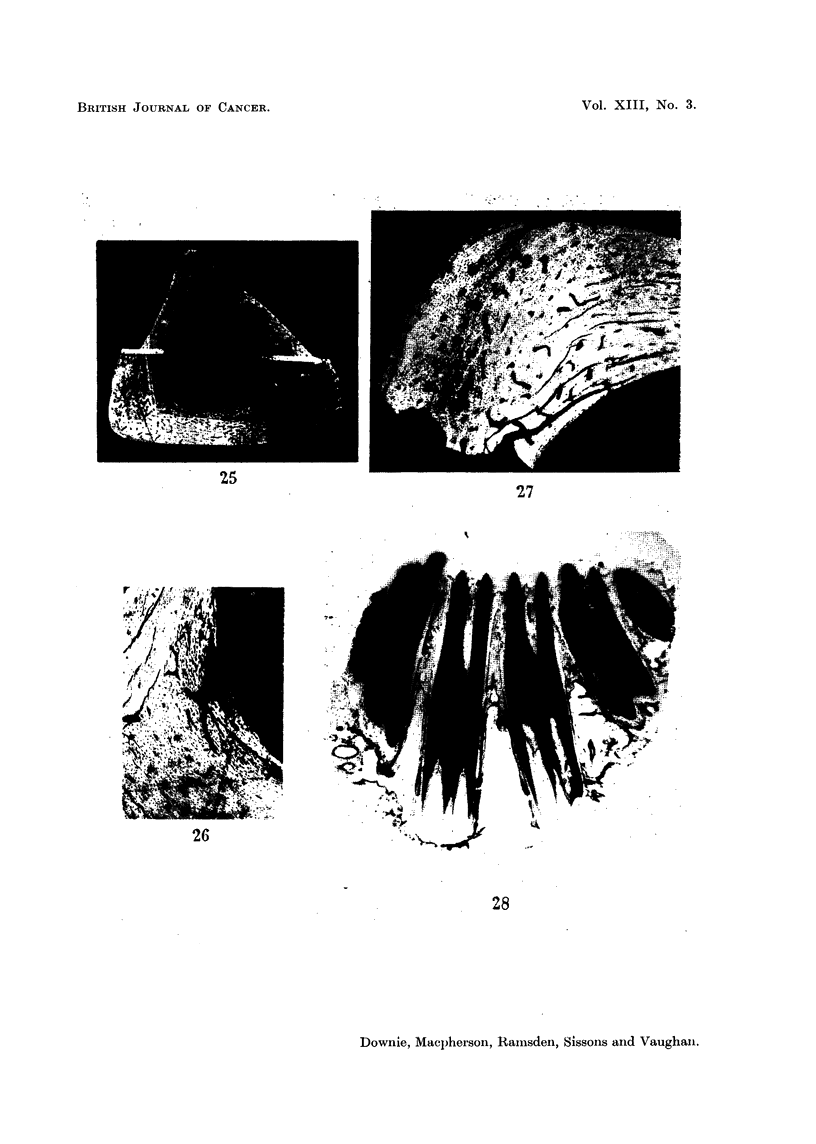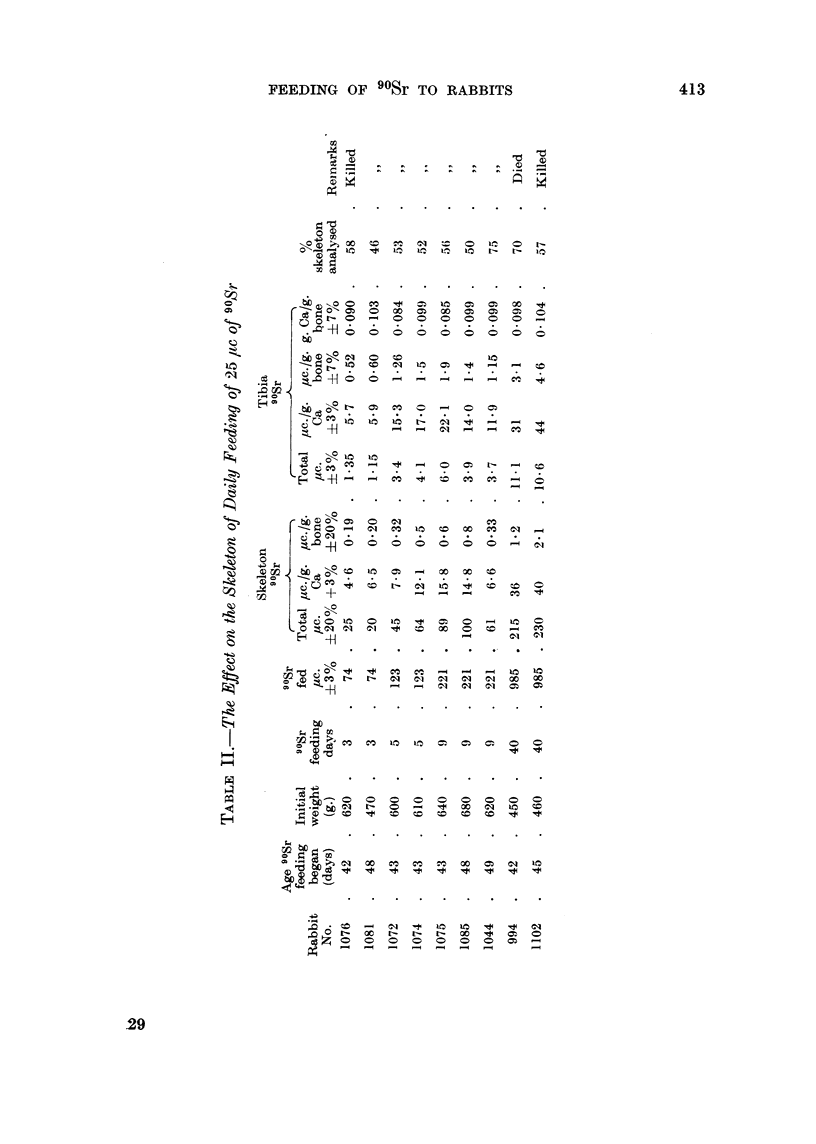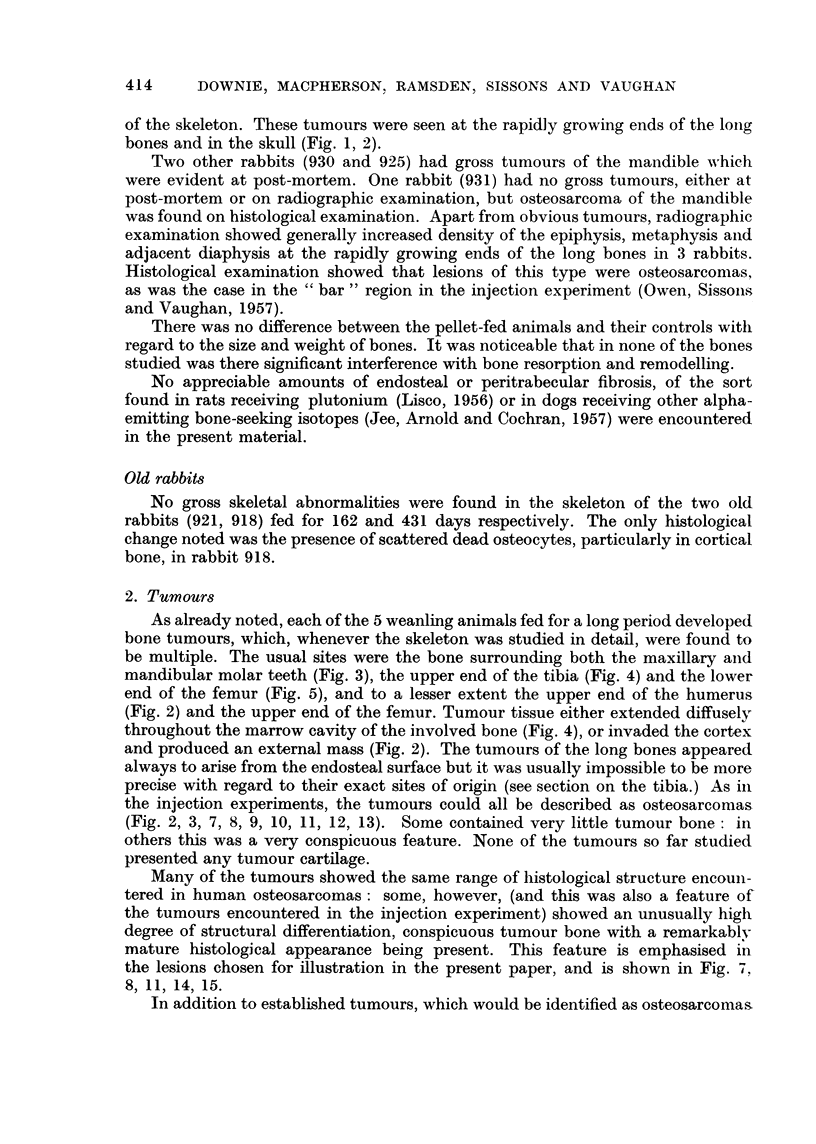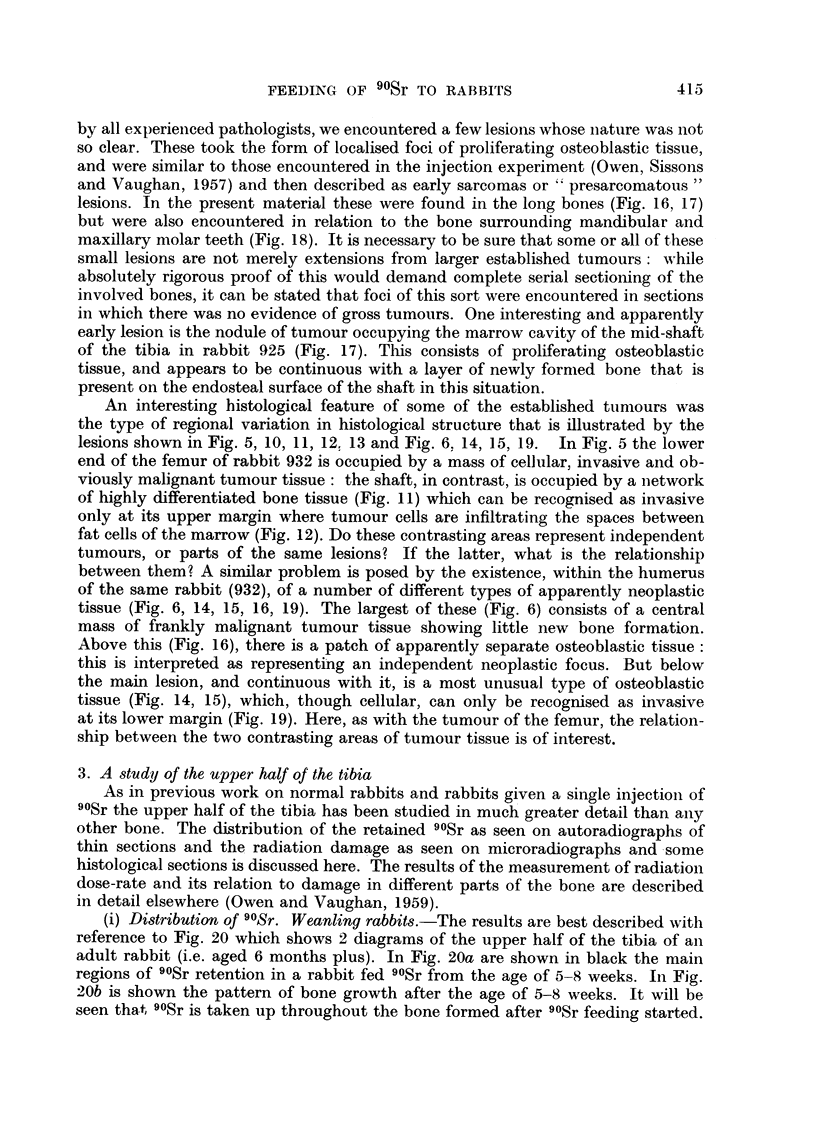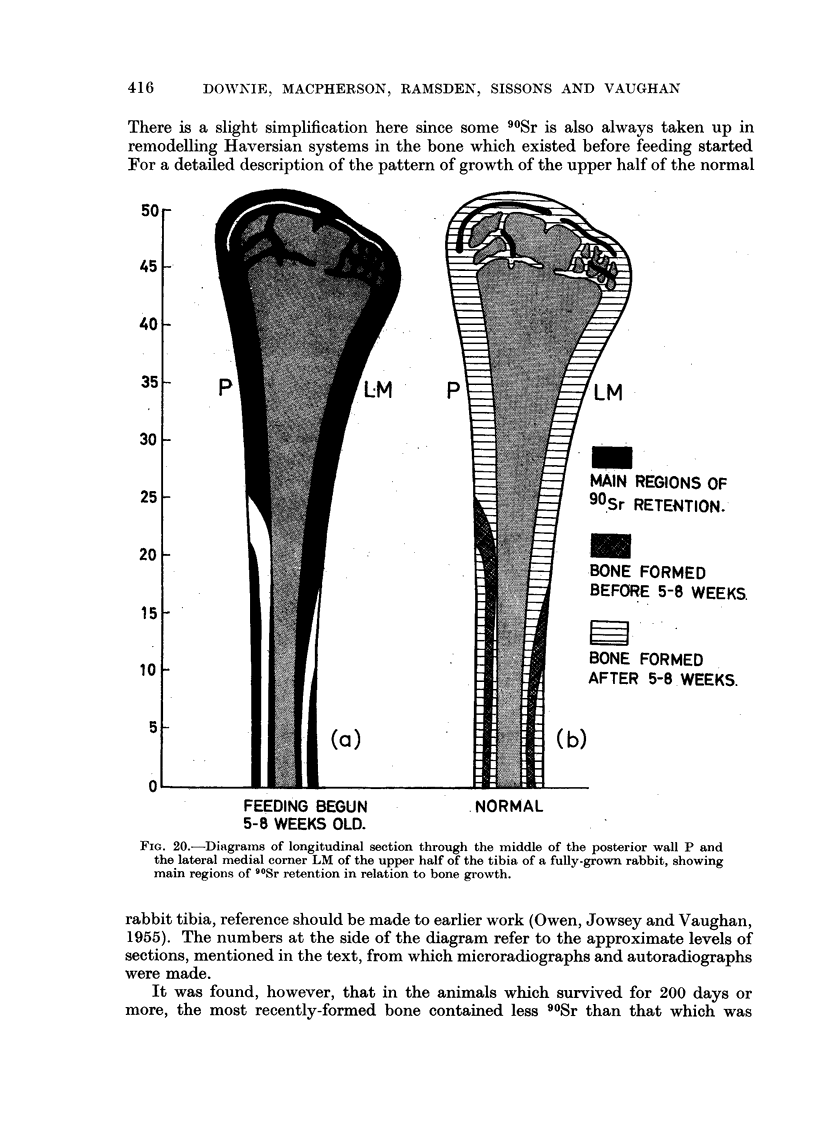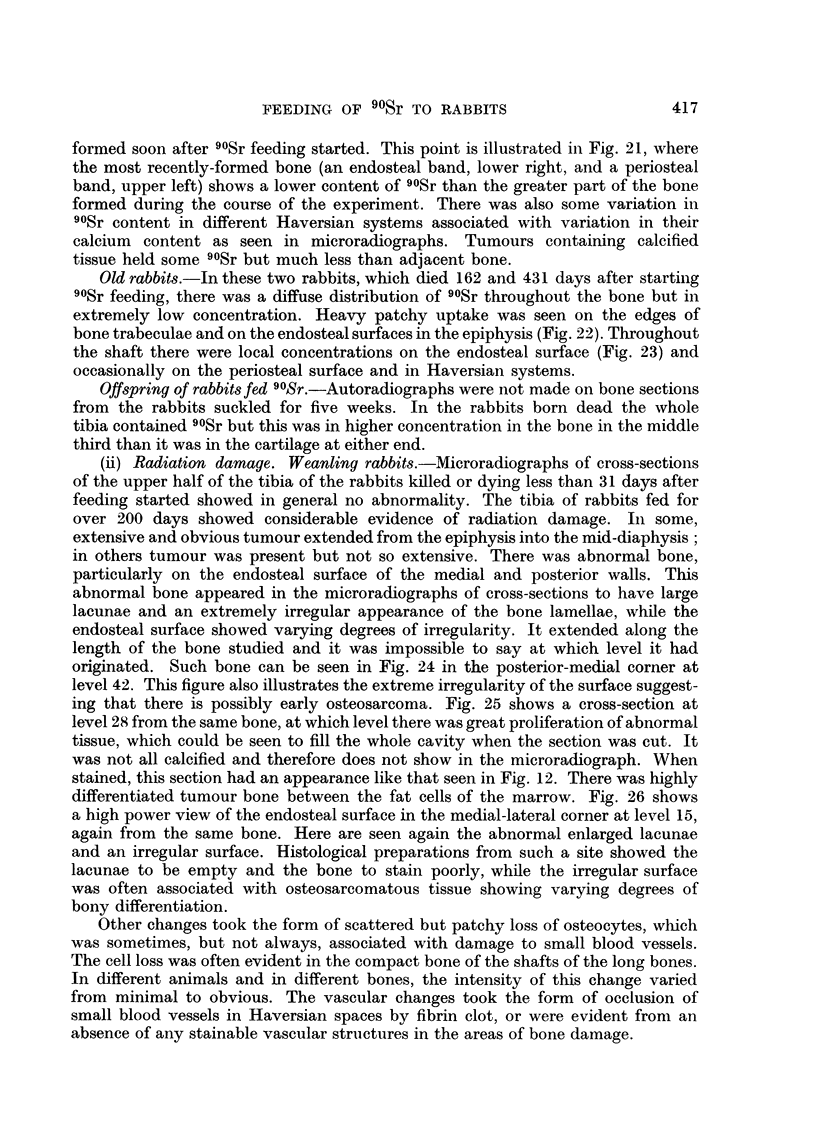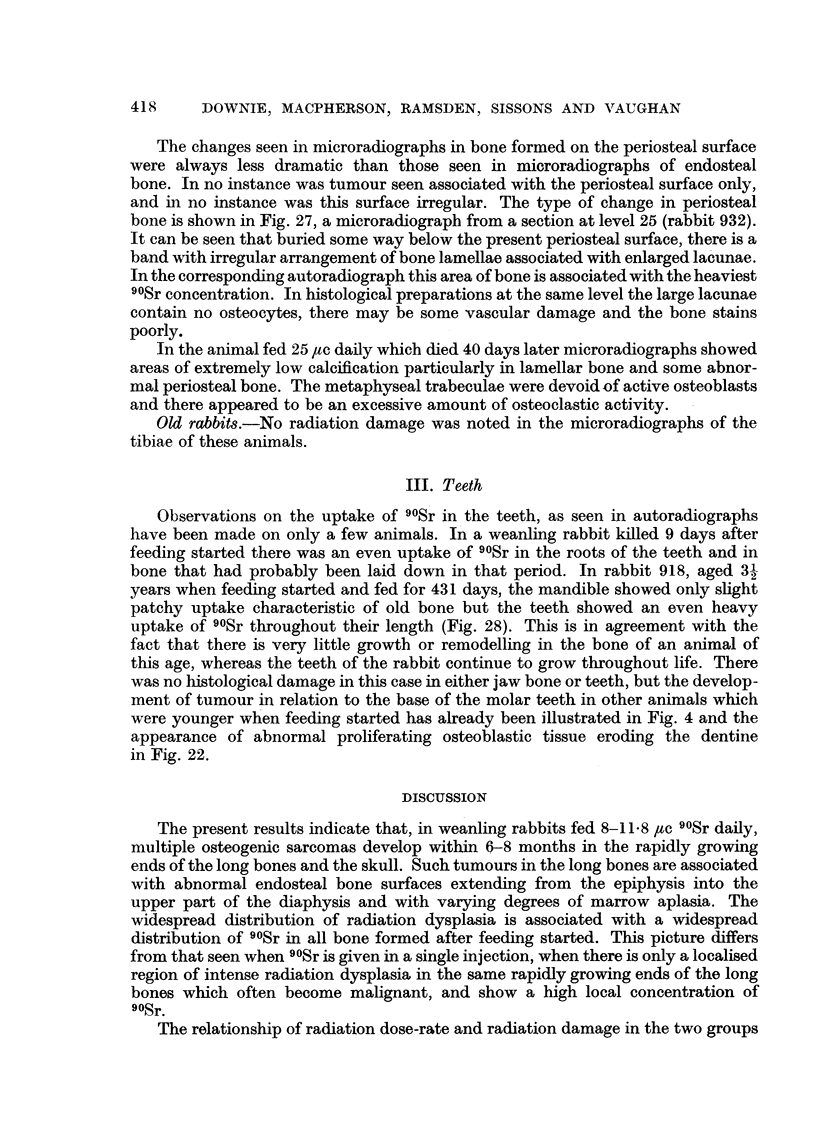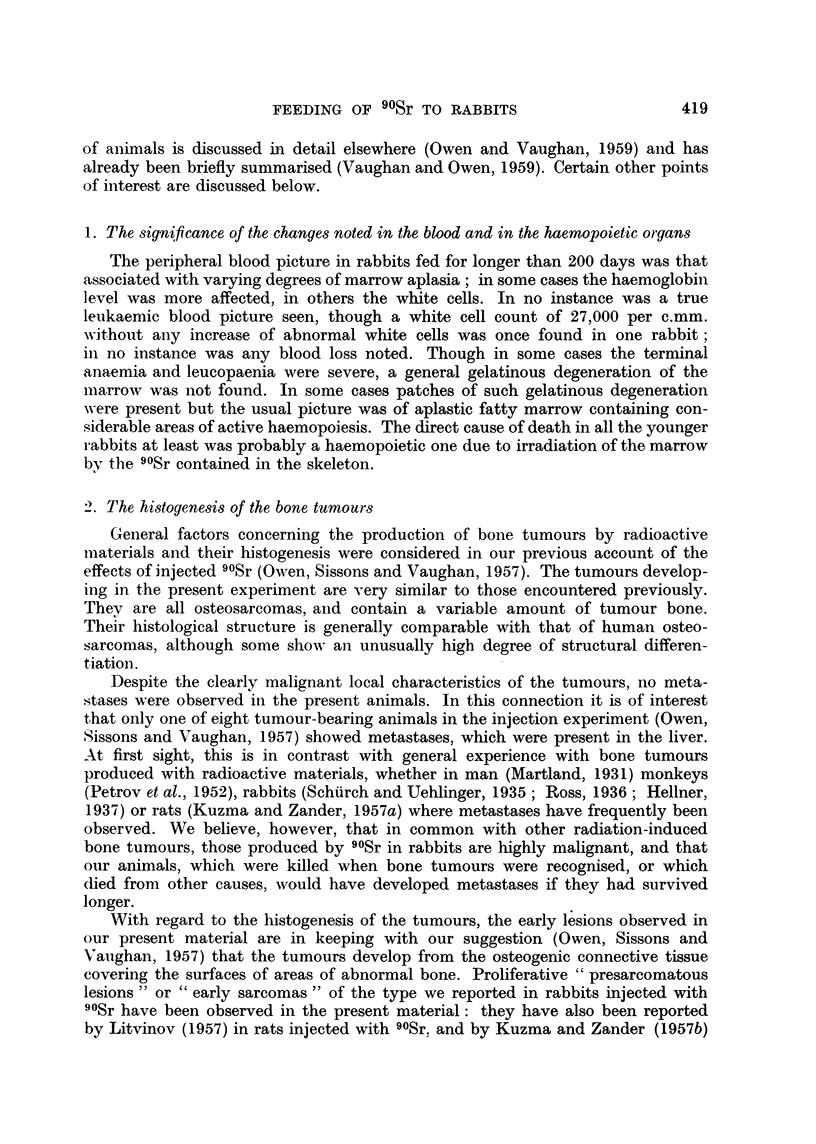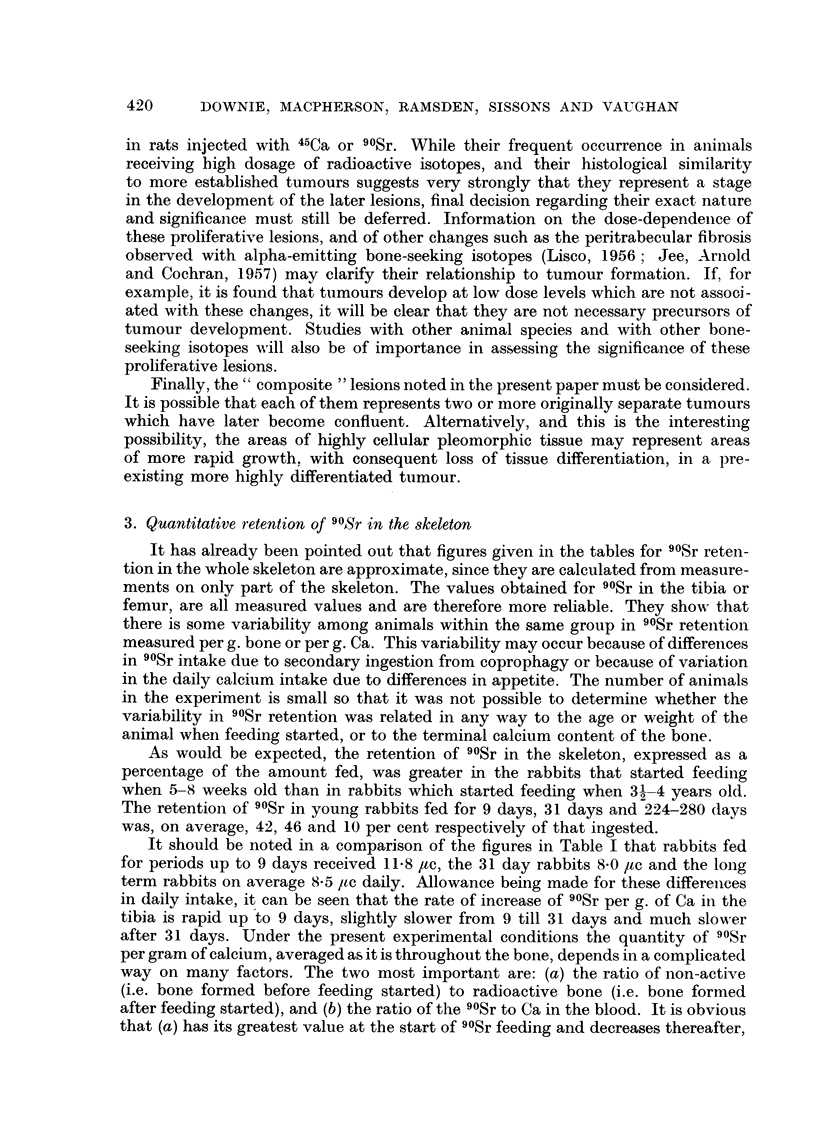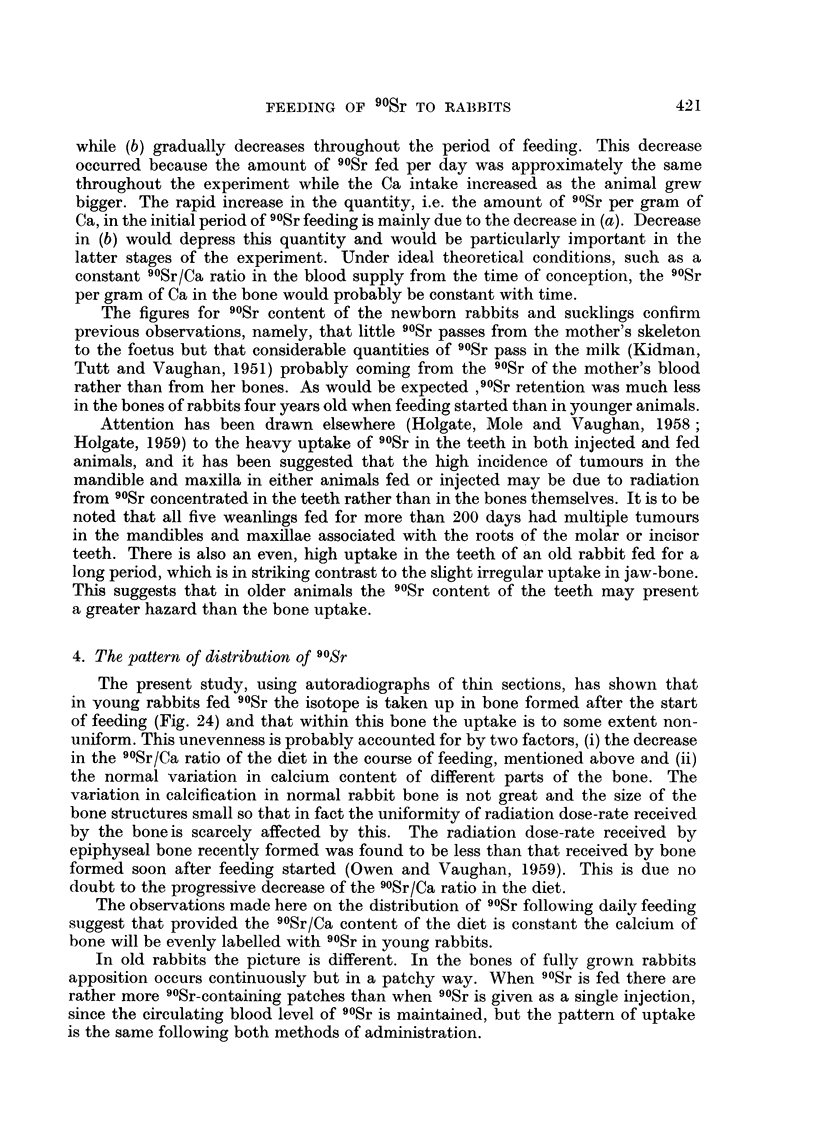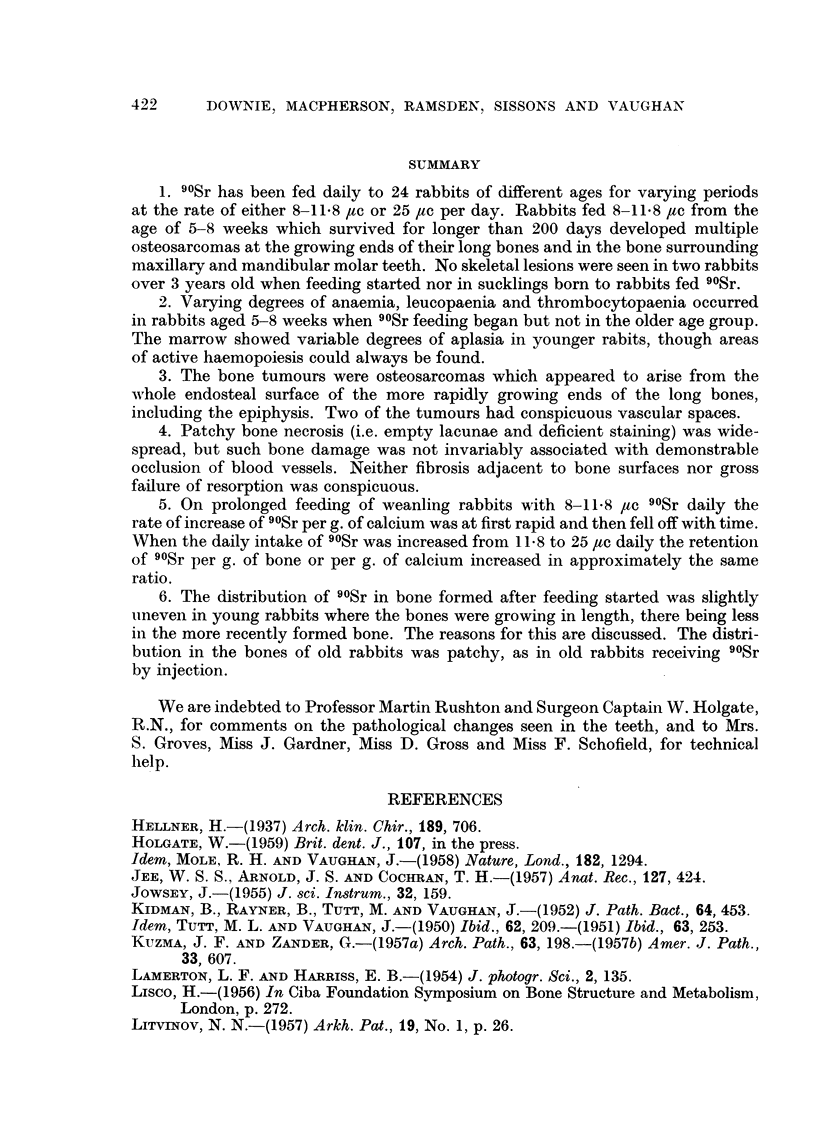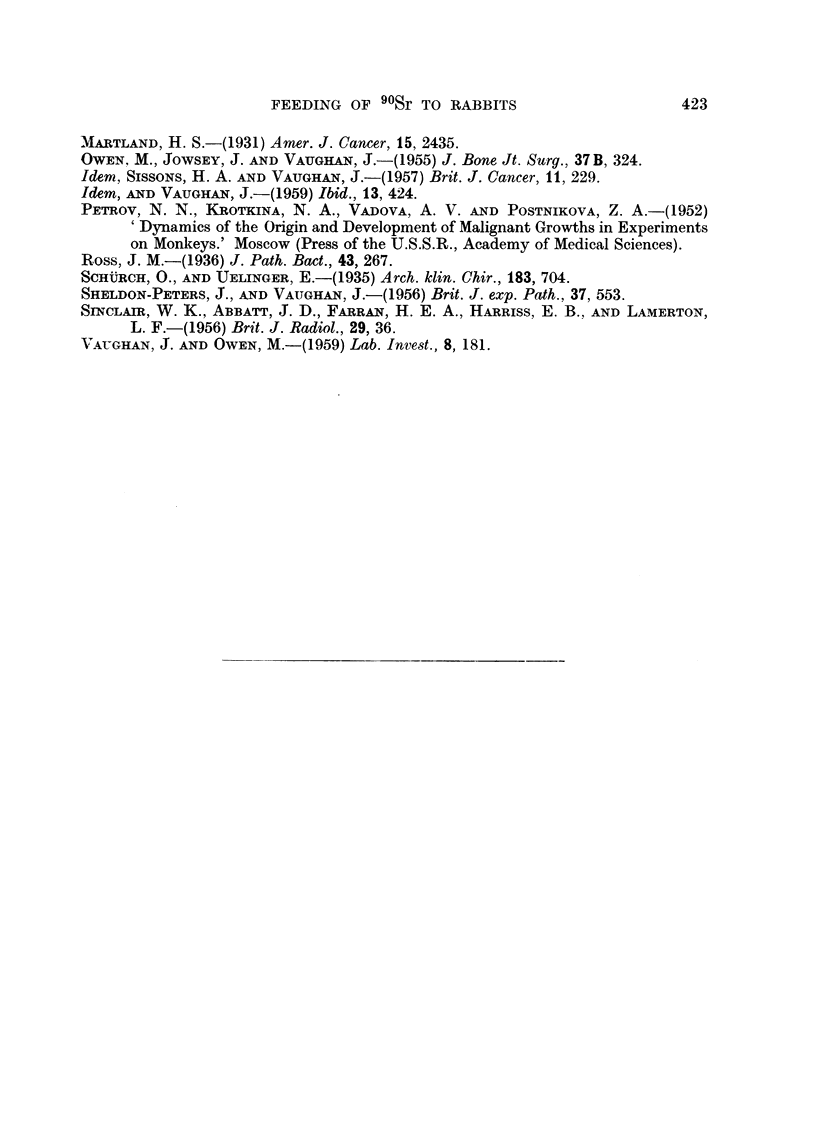# The Effect of Daily Feeding of ^90^Sr to Rabbits

**DOI:** 10.1038/bjc.1959.47

**Published:** 1959-09

**Authors:** Eileen D. Downie, Sheila Macpherson, Eileen N. Ramsden, H. A. Sissons, Janet Vaughan

## Abstract

**Images:**


### THE EFFECT OF DAILY FEEDING OF $^{90}Sr$ TO RABBITS

EILEEN D. DOWNIE, SHEILA MACPHERSON, EILEEN N. RAMSDEN, H. A. SISSONS AND JANET VAUGHAN

*From the Medical Research Council Group for Research on Bone Seeking Isotopes, Churchill Hospital, Oxford, and the Institute for Orthopaedics, London*

Received for publication June 30, 1959

PREVIOUS papers have discussed the amount of $^{90}Sr$ retained in the skeleton of rabbits following a single injection of this isotope and the pattern of its distribution (Kidman, Tutt and Vaughan, 1950, 1951 ; Kidman, Rayner, Tutt and Vaughan, 1952 ; Owen, Sissons and Vaughan, 1957). The present paper both describes the effects of continuous feeding of $^{90}Sr$ to rabbits and analyses the pattern and amount of isotope retention. A comparison of the damage produced by the two methods of administration and its relation to radiation dose-rate and accumulated dose will be discussed elsewhere (Owen and Vaughan, 1959).

### EXPERIMENTAL METHODS

### 1. *Rabbits*

Dutch rabbits of the same stock and on the same diet as those in previous experiments were used for $^{90}Sr$ feeding (Owen, Sissons and Vaughan, 1957). This diet gave a calcium intake of 0·28 g. daily (expressed as calcium oxide) when feeding started and of 0·50 g. when the animals were 6 months old (Kidman, Tutt and Vaughan, 1950). Twenty-two of the rabbits were weanlings, i.e. 5–8 weeks old when feeding started. Two in this age group had litters during the experimental period. Four rabbits born dead from $^{90}Sr$-fed mothers and two rabbits suckled for five weeks by their $^{90}Sr$-fed mothers were included. Two rabbits were over 3 years old when feeding started. Some of the rabbits were killed, others died during the period of $^{90}Sr$ feeding. At autopsy the skeletons were dissected, weighed and in most cases radiographed. Various bones were taken for special studies described below. Knowing the weight, it was possible to allow for any bone so used in making estimations of total skeletal retention of $^{90}Sr$. A variety of soft tissues including representative samples of the various parts of the intestinal tract, as well as liver, spleen, kidneys and gonads, were examined histologically.

### 2. *Method of feeding $^{90}Sr$*

$^{90}Sr$ was fed incorporated in an M.R.C. pellet. The animals were starved overnight and fed a $^{90}Sr$ pellet each morning to ensure ingestion. In addition to the $^{90}Sr$ fed in the pellet, there was what might be termed secondary ingestion, due to the rabbit's habit of coprophagy. Intake from this is not included in the $^{90}Sr$ intake figures.

It would theoretically have been more satisfactory to have given the rabbits all their food in the form of pellets containing $^{90}Sr$ so that the $^{90}Sr$/Ca ratio in the diet could have been kept constant throughout the experiment. The pellet diet, however, has a higher calcium content than the oats, hay and greenstuff diet which had been given in other experiments (Kidman *et al.*, 1950 ; Owen, Jowsey and Vaughan, 1955 ; Owen, Sissons and Vaughan, 1957), and the experimental results obtained here would not therefore have been comparable.

3. *Preparation of $^{90}$Sr pellets*

A known weight and number of M.R.C. pellets was moistened with distilled water. To this was added a solution of $^{90}$Sr in 0·1 N HCl which was calculated to give 10 $\mu$c $^{90}$Sr per pellet. The paste was then made into pellets. Although every effort was made to mix the paste thoroughly and to make the pellets to constant size there was inevitably some slight variation. For instance a sample of 15 pellets from one batch had a mean value of 8·5 $\mu$c per pellet, while a sample of 20 pellets from another batch had a mean value of 11·8 $\mu$c per pellet. The other batches fell between 8·0 and 11·8 $\mu$c per pellet. A further batch of pellets was made up using a solution of higher activity which gave a mean content of 25 $\mu$c. It was known which batch was fed to each rabbit so that the mean value of the batch was taken into account in calculating total intake.

4. *Estimation of $^{90}$Sr and calcium*

The bones were dissolved in nitric acid, and $^{90}$Sr retention measured by methods described elsewhere (Kidman, Tutt and Vaughan, 1950). Aliquots equivalent to about 0·03 g. bone were evaporated to dryness and ashed, and their calcium content estimated by the method already described (Sheldon-Peters and Vaughan, 1956).

The figure for $^{90}$Sr fed was calculated from the $^{90}$Sr content of the pellets and the number of pellets eaten from each batch. The $^{90}$Sr content of the pellets was known to within $\pm$ 3 per cent, except when the number of pellets fed was less than 5, when the error became $\pm$ 10 per cent. Since the whole skeleton could not be used for $^{90}$Sr estimation, the figures for its total retention was calculated from the measured amount in those parts available. The percentage analysed is stated.

The figures at the top of each column in the tables are standard deviations on the analytical methods with the exceptions of those involving the weights of tibiae or skeletons. The difficulty of weighing wet bones reproducibly makes the results in these columns reliable within an estimated range of $\pm$ 7 per cent in the case of tibiae and $\pm$ 20 per cent in the case of skeletons.

5. *Haematological and histological techniques*

Haematological and histological techniques were those used in previous work (Owen, Sissons and Vaughan, 1957). Platelets were estimated from examination of stained films. The standard deviation on the haemoglobin value of normal rabbits in the younger age group in this laboratory is 0·88 and on the total white cell count 1560. In the older age group the haemoglobin standard deviation is 0·65 and for the white count 957.

6. *The use of the tibia*

Since the pattern of growth has been studied in more detail in the tibia than in any other bone (Owen, Jowsey and Vaughan, 1955), the two tibiae of each rabbit were used for several different purposes. The right tibia was first used for radiation dosimetry measurements which are reported in detail elsewhere (Owen and Vaughan, 1959). For this purpose it was necessary to cut the proximal half of the bone into two longitudinal halves for the preparation of contact autoradiographs needed for dosimetry measurements (Lamerton and Harriss, 1954; Sinclair, Abbatt, Farran, Harriss and Lamerton, 1956). The two halves were then stuck together and thin cross-sections cut for the preparation of autoradiographs and microradiographs. The left tibia was used either for chemical analysis of $^{90}$Sr and Ca content or for histological examination.

7. *Microradiographs and autoradiographs*

Microradiographs and autoradiographs were prepared from thin cross-sections of the tibia and from the mandible, as previously described (Jowsey, 1955; Owen, Jowsey and Vaughan, 1955). Kodak Scientific Plate autoradiographic stripping film (AR10) was used except when the low $^{90}$Sr content of bone made the use of Kodak Scientific Plate fast autoradiographic stripping film (AR50) necessary.

### RESULTS

### I. *General Effects of $^{90}$Sr*

The effect of feeding 8–11·8 $\mu$c $^{90}$Sr daily on the weight and the blood picture of 8 rabbits that were 5–8 weeks old when feeding started, and of two much older rabbits, are shown in Table I, together with the amount of $^{90}$Sr fed and the amount retained in the skeleton in 14 rabbits and in six progeny born during the exerimental period. Figures for the amount of $^{90}$Sr fed and retained in 9 rabbits fed 25 $\mu$c daily are shown in Table II.

*Soft Tissues*

Sections of lungs, liver, spleen, kidneys, adrenals and gonads, and of the walls of various parts of the alimentary canal, failed to show histological evidence of any important radiation damage. In some instances, post-mortem changes were present and may have obscured any minor abnormalities of the alimentary mucous membranes, but neither frank ulceration nor scarring of the intestinal wall was encountered. In rabbits dying in the first few weeks of $^{90}$Sr feeding, histological evidence of damage to bone marrow was slight, being restricted to some loss of haemopoietic cells in the metaphyseal regions of the long bones. In rabbits dying later in the experiment, i.e. more than 200 days after feeding started, bone marrow was fatty and atrophic although areas of active haemopoiesis could always be found. Mucoid degeneration of bone marrow was found in a small area in only one animal (925).

### II. *Effects on the Skeleton*

1. *General*

*Weanling rabbits fed 8–11·8 $\mu$c daily.*—No gross skeletal abnormalities were noted in the skeletons of weanling rabbits fed for 3 to 48 days. Gross skeletal abnormalities were observed in 4 (922, 925, 930, 932) of the 5 rabbits which survived more than 200 days from the time feeding started at 5–8 weeks.

Two of these rabbits (922 and 932) developed multiple bone tumours which were evident by general inspection during life, and by radiographic examination

TABLE I.—*The Effect on the Skeleton, Haemoglobin, White Blood Count, Platelets and Weight of Daily Feeding of* 8–11·8 *μc* $^{90}$*Sr*

| Rabbit No. | Age $^{90}$Sr feeding began (days) | Initial weight (g.) | $^{90}$Sr feeding days | $^{90}$Sr fed μc. ±3%* | Skeleton $^{90}$Sr: Total μc.* ±20% | Skeleton $^{90}$Sr: μc./g. Ca* ±3% | Skeleton $^{90}$Sr: μc./g. bone ±20%* | Tibia $^{90}$Sr: Total μc. ±3%* | Tibia $^{90}$Sr: μc./g. Ca ±3%* | Tibia $^{90}$Sr: μc./g. bone ±7%* | Tibia $^{90}$Sr: g. Ca/g. bone* ±7% | % skeleton analysed | W.B.C. (thousands c.mm.): Before $^{90}$Sr feeding | W.B.C. (thousands c.mm.): Terminal | Hbg. %: Before $^{90}$Sr feeding | Hbg. %: Terminal | Platelets | Wt. loss (g.) | Remarks |
|---|---|---|---|---|---|---|---|---|---|---|---|---|---|---|---|---|---|---|---|
| 1108 | 45 | 620 | 3 | 35 | 19 | 2·9 | 0·17 | 0·71 | 2·9 | 0·29 | 0·103 | 54 | — | — | — | — | .. | — | Killed |
| 1110 | 46 | 690 | 5 | 59 | 17 | 3·0 | 0·14 | 1·08 | 3·1 | 0·26 | 0·084 | 75 | — | — | — | — | .. | — | ,, |
| 1112 | 46 | 620 | 5 | 59 | 13 | 2·4 | 0·12 | 1·08 | 3·8 | 0·38 | 0·102 | 71 | — | — | — | — | .. | — | ,, |
| 1109 | 45 | 740 | 9 | 106 | 25 | 3·3 | 0·18 | 1·57 | 7·5 | 0·46 | 0·061 | 75 | — | — | — | — | .. | — | ,, |
| 1111 | 45 | 620 | 9 | 106 | 24 | 5·1 | 0·19 | 1·46 | 7·7 | 0·52 | 0·67 | 87 | — | — | — | — | .. | — | ,, |
| 973 | 44 | 650 | 31 | 248 | 130 | 12·9 | 1·1 | 4·5 | 10·8 | 1·6 | 0·15 | 45 | 4600 | 3700 | 12·6 | 11·1 | .. | 0 | Died (accident) |
| 974 | 44 | 550 | 31 | 248 | 100 | 9·3 | 0·8 | 3·0 | 6·9 | 1·0 | 0·15 | 55 | 5100 | 6400 | 13·3 | 13·3 | .. | 0 | Killed |
| 924 | 52 | 810 | 48 | 440 | — | — | — | — | — | — | — | — | 5000 | 4600 | 12·0 | 11·8 | R | 0 | ,, |
| 930 | 52 | 730 | 224 | 1870 | 220 | 14·0 | 1·1 | 12·3 | 15·6 | 2·7 | 0·18 | 95 | 6600 | 6800 | 13·0 | 5·8 | ,, | 50 | Died |
| 931† | 54 | 780 | 240 | 2020 | 150 | 8·8 | 0·9 | 9·6 | 11·7 | 2·0 | 0·17 | 75 | 5500 | 2600 | 13·8 | 11·2 | ,, | 50 | Killed |
| 925‡ | 54 | 890 | 252 | 2060 | 190 | 10·6 | 1·3 | 12·4 | 13·5 | 2·7 | 0·20 | 70 | 4200 | 1700 | 12·4 | 4·6 | ,, | 730 | Died |
| 932 | 52 | 730 | 263 | 2060 | 220 | 13·2 | 1·1 | 9·2F | 6·9 | 1·5 | 0·21 | 60 | 4000 | 1700 | 13·3 | 13·0 | S | 10 | ,, |
| 922 | 54 | 890 | 280 | 2070 | 250 | 13·0 | 1·5 | 13·0F | 13·5 | 2·2 | 0·16 | 50 | 3100 | 2000 | 11·8 | 8·8 | R | 620 | ,, |
| 925*a* | Foetus | .. | .. | .. | 0·23 | 0·81 | 0·029 | 0·0066 | 0·95 | 0·077 | .. | .. | .. | .. | .. | .. | .. | .. | Born dead |
| 925*b* | ,, | .. | .. | .. | 0·18 | 0·90 | 0·019 | 0·0074 | 0·96 | 0·074 | .. | .. | .. | .. | .. | .. | .. | .. | ,, ,, |
| 931*a* | ,, | .. | .. | .. | 0·20 | 0·85 | 0·028 | 0·0047 | 0·52 | 0·060 | .. | .. | .. | .. | .. | .. | .. | .. | ,, ,, |
| 931*b* | ,, | .. | .. | .. | 0·19 | 0·91 | 0·025 | 0·0044 | 0·61 | 0·051 | .. | .. | .. | .. | .. | .. | .. | .. | ,, ,, |
| 931*c* | 0 | .. | 35 | .. | 8·9 | 1·75 | 0·10 | 0·49 | 1·95 | 0·23 | .. | .. | .. | .. | .. | .. | .. | .. | Killed |
| 931*d* | 0 | .. | 35 | .. | 10·5 | 1·96 | 0·10 | 0·61 | 2·13 | 0·25 | .. | .. | .. | .. | .. | .. | .. | .. | ,, |
| 921 | 4 years | .. | 162 | 1300 | 8·1 | 0·35 | 0·031 | 0·126 | 0·126 | 0·022 | 0·17 | 95 | 4400 | 8600§ | 10·9 | 10·5 | S | 12 | Died |
| 918 | 3½ years | .. | 431 | 6750° | 130 | .. | .. | 2·00 | 1·99 | 0·33 | 0·17 | 2 | 5700 | 2400 | 11·8 | 11·0 | ,, | 6 | Killed |

F = femur.
† = had a litter, 2 dead, 2 suckled 5 weeks : *a, b, c, d*
‡ = had a litter, 2 dead : *a, b.*
R = reduced.
S = steady level.

° This rabbit received some pellets containing 25 μc. $^{90}$Sr.

§ rose as high as 26,000.

* see page 3.

### EXPLANATION OF PLATES

FIG. 1.—Rabbit 932, fed $^{90}Sr$ for 263 days from the age of six weeks—radiograph. Note : (i) tumour of upper end of left tibia associated with increased density ; (ii) tumour of mandible.

FIG. 2.—Rabbit 922, a longitudinal section from the upper part of the humerus. Tumour tissue is present in both the epiphysis and metaphysis, and projects as a large subperiosteal mass. The tumour is an osteosarcoma, with relatively little bone formation. $\times$ 3.

FIG. 3.—Rabbit 922, transverse section of skull, showing tumour tissue surrounding the roots of the maxillary molar teeth on each side. The lesions are osteosarcomas, and appear to have had origin in the bone of the tooth sockets. $\times 1\frac{1}{4}$.

FIG. 4.—Rabbit 922, a longitudinal section of the upper part of the tibia, showing complete replacement of marrow by bony tumour tissue. The posterior cortex is invaded in several places, and a subperiosteal extension is present. $\times$ 3.

FIG. 5.—Rabbit 932, a longitudinal section of the lower part of the femur. Tumour tissue occupies the lower end of the bone and appears to be extending upwards in the marrow cavity. Note the contrasting appearance of the tumour tissue in different parts of the lesion. $\times 1\frac{3}{4}$.

FIG. 6.—Rabbit 932, a longitudinal section from the upper part of the humerus. The marrow cavity is occupied by a mass of cellular tumour tissue containing some vascular cysts. The lesion is an osteosarcoma with inconspicuous bone formation. $\times 1\frac{3}{4}$.

FIG. 7.—Rabbit 922, the highly differentiated osteoblastic tumour tissue comprising the subperiosteal mass seen in Fig. 4. $\times$ 43.

FIG. 8.—Rabbit 922, ossifying tumour tissue occupying marrow spaces from the upper part of the lesion seen in Fig. 4. $\times$ 43.

FIG. 9.—Rabbit 922, pleomorphic tissue from the advancing margin of the tumour seen in Fig. 4. $\times$ 43.

FIG. 10.—Rabbit 932, cellular tissue with inconspicuous strands of tumour bone from the lower part of the lesion shown in Fig. 5. $\times$ 63.

FIG. 11.—Rabbit 932, highly differentiated tumour bone forming a network between fat cells of the marrow, from the upper part of the lesion shown in Fig. 5. $\times$ 33.

FIG. 12.—Rabbit 932, the upper margin of the lesion shown in Fig. 5, tumour cells are extending between the fat cells of the marrow and are forming a network of tumour bone which appears as darkly stained material in the lower part of the field. $\times$ 30.

FIG. 13.—Rabbit 932, one of the nodular masses of cellular tumour tissue from the central part of the lesion in Fig. 5. $\times$ 30.

FIG. 14.—Rabbit 932, unusual new bone formation (? highly differentiated tumour tissue) continuous with the lower part of the lesion shown in Fig. 6. $\times$ 30.

FIG. 15.—Rabbit 932, another field showing the more detailed structure of tissue similar to that shown in Fig. 13. $\times$ 56.

FIG. 16.—Rabbit 932, part of an area of active bone formation (? early tumour) which is evident in the metaphysis in Fig. 5 above and separate from the main lesion. $\times$ 43.

FIG. 17.—Rabbit 925, longitudinal section of mid-shaft of tibia, showing a mass of osteoblastic tumour tissue which appears to be continuous with a layer of newly formed bone on the endosteal surface of the shaft. $\times$ 9.

FIG. 18.—Rabbit 922, section through the base of a mandibular molar tooth, showing erosion of dentine by a localised area of proliferating osteoblastic tissue (? early tumour). $\times$ 45.

FIG. 19.—Rabbit 932, the lower margin of the unusual bone tissues shown in Fig. 14 and 15. Note the resemblance to Fig. 12. $\times$ 57.

FIG. 21.—Rabbit 932, fed $^{90}Sr$ for 263 days from the age of six weeks. Section 17. Autoradiograph. Note : (i) uneven uptake of $^{90}Sr$ in bone formed after feeding started, the more recently formed bone on the endosteal and periosteal surfaces containing less $^{90}Sr$. (ii) High $^{90}Sr$ content of certain Haversian systems. (iii) Abnormal endosteal bone in lower right-hand corner. $\times$ 5.

FIG. 22.—Rabbit 921, fed $^{90}Sr$ for 162 days from the age of 4 years. Section 47—upper end of tibia. Autoradiograph. Note heavy uptake of $^{90}Sr$ on edges of trabeculae, patchy in distribution. $\times$ 40.

FIG. 23.—Rabbit 921, fed $^{90}Sr$ for 162 days from the age of 4 years. Section 19—upper half of tibia. Autoradiograph. Note: (i) heavy, though patchy, uptake of $^{90}Sr$ on endosteal surfaces. (ii) Occasional heavy periosteal uptake of $^{90}Sr$.

FIG. 24.—Rabbit 932, fed $^{90}Sr$ for 263 days from the age of six weeks. Section 42 upper half of tibia ; microradiograph of posterior-medial corner. Note : abnormal bone on endosteal surface, possible osteosarcoma. $\times$ 21.

FIG. 25.—Rabbit 932, fed $^{90}Sr$ for 263 days from the age of six weeks. Section 28—upper half of tibia ; microradiograph. Note : (i) osteosarcoma extending along part of posterior, medial and lateral endosteal surface. (ii) The bands of white are the sites where the bone was cut to prepare gross autoradiographs. Compare with Fig. 11. $\times$ 6.

FIG. 26.—Rabbit 932, fed $^{90}Sr$ for 263 days from the age of six weeks. Section 15—upper half of tibia, microradiograph of lateral-medial corner. Note : abnormal endosteal bone, large lacunae, irregular lamellae, irregular surface. $\times$ 24.

FIG. 27.—Rabbit 932, fed $^{90}Sr$ for 263 days from the age of six weeks. Section 25—upper half of tibia ; microradiograph. Note : abnormal buried periosteal bone showing large lacunae and irregular arrangements of the lamellae. $\times$ 22.

FIG. 28.—Rabbit 918, fed $^{90}Sr$ for 431 days from the age of 3·5 years. Autoradiograph of molar teeth and adjacent mandible. Note relatively low $^{90}Sr$ uptake in bone and high uptake throughout teeth. $\times$ 4.

1

2

3

4

5

6

7

8

9

10

11

12

13

14

15

16

17

Downie, Macpherson, Ramsden, Sissons and Vaughan.

18

19

21

22

23

24

25

27

26

28

Downie, Macpherson, Ramsden, Sissons and Vaughan.

TABLE II.—*The Effect on the Skeleton of Daily Feeding of 25 μc of $^{90}Sr$*

| Rabbit No. | Age $^{90}Sr$ feeding began (days) | Initial weight (g.) | $^{90}Sr$ feeding days | $^{90}Sr$ fed μc. ±3% | Skeleton $^{90}Sr$ Total μc. ±20% | Skeleton $^{90}Sr$ μc./g. Ca +3% | Skeleton $^{90}Sr$ μc./g. bone ±20% | Tibia $^{90}Sr$ Total μc. ±3% | Tibia $^{90}Sr$ μc./g. Ca ±3% | Tibia $^{90}Sr$ μc./g. bone ±7% | Tibia $^{90}Sr$ g. Ca/g. bone ±7% | % skeleton analysed | Remarks |
|---|---|---|---|---|---|---|---|---|---|---|---|---|---|
| 1076 | 42 | 620 | 3 | 74 | 25 | 4·6 | 0·19 | 1·35 | 5·7 | 0·52 | 0·090 | 58 | Killed |
| 1081 | 48 | 470 | 3 | 74 | 20 | 6·5 | 0·20 | 1·15 | 5·9 | 0·60 | 0·103 | 46 | ,, |
| 1072 | 43 | 600 | 5 | 123 | 45 | 7·9 | 0·32 | 3·4 | 15·3 | 1·26 | 0·084 | 53 | ,, |
| 1074 | 43 | 610 | 5 | 123 | 64 | 12·1 | 0·5 | 4·1 | 17·0 | 1·5 | 0·099 | 52 | ,, |
| 1075 | 43 | 640 | 9 | 221 | 89 | 15·8 | 0·6 | 6·0 | 22·1 | 1·9 | 0·085 | 56 | ,, |
| 1085 | 48 | 680 | 9 | 221 | 100 | 14·8 | 0·8 | 3·9 | 14·0 | 1·4 | 0·099 | 50 | ,, |
| 1044 | 49 | 620 | 9 | 221 | 61 | 6·6 | 0·33 | 3·7 | 11·9 | 1·15 | 0·099 | 75 | ,, |
| 994 | 42 | 450 | 40 | 985 | 215 | 36 | 1·2 | 11·1 | 31 | 3·1 | 0·098 | 70 | Died |
| 1102 | 45 | 460 | 40 | 985 | 230 | 40 | 2·1 | 10·6 | 44 | 4·6 | 0·104 | 57 | Killed |

of the skeleton. These tumours were seen at the rapidly growing ends of the long bones and in the skull (Fig. 1, 2).

Two other rabbits (930 and 925) had gross tumours of the mandible which were evident at post-mortem. One rabbit (931) had no gross tumours, either at post-mortem or on radiographic examination, but osteosarcoma of the mandible was found on histological examination. Apart from obvious tumours, radiographic examination showed generally increased density of the epiphysis, metaphysis and adjacent diaphysis at the rapidly growing ends of the long bones in 3 rabbits. Histological examination showed that lesions of this type were osteosarcomas, as was the case in the " bar " region in the injection experiment (Owen, Sissons and Vaughan, 1957).

There was no difference between the pellet-fed animals and their controls with regard to the size and weight of bones. It was noticeable that in none of the bones studied was there significant interference with bone resorption and remodelling.

No appreciable amounts of endosteal or peritrabecular fibrosis, of the sort found in rats receiving plutonium (Lisco, 1956) or in dogs receiving other alpha-emitting bone-seeking isotopes (Jee, Arnold and Cochran, 1957) were encountered in the present material.

*Old rabbits*

No gross skeletal abnormalities were found in the skeleton of the two old rabbits (921, 918) fed for 162 and 431 days respectively. The only histological change noted was the presence of scattered dead osteocytes, particularly in cortical bone, in rabbit 918.

2. *Tumours*

As already noted, each of the 5 weanling animals fed for a long period developed bone tumours, which, whenever the skeleton was studied in detail, were found to be multiple. The usual sites were the bone surrounding both the maxillary and mandibular molar teeth (Fig. 3), the upper end of the tibia (Fig. 4) and the lower end of the femur (Fig. 5), and to a lesser extent the upper end of the humerus (Fig. 2) and the upper end of the femur. Tumour tissue either extended diffusely throughout the marrow cavity of the involved bone (Fig. 4), or invaded the cortex and produced an external mass (Fig. 2). The tumours of the long bones appeared always to arise from the endosteal surface but it was usually impossible to be more precise with regard to their exact sites of origin (see section on the tibia.) As in the injection experiments, the tumours could all be described as osteosarcomas (Fig. 2, 3, 7, 8, 9, 10, 11, 12, 13). Some contained very little tumour bone : in others this was a very conspicuous feature. None of the tumours so far studied presented any tumour cartilage.

Many of the tumours showed the same range of histological structure encountered in human osteosarcomas : some, however, (and this was also a feature of the tumours encountered in the injection experiment) showed an unusually high degree of structural differentiation, conspicuous tumour bone with a remarkably mature histological appearance being present. This feature is emphasised in the lesions chosen for illustration in the present paper, and is shown in Fig. 7, 8, 11, 14, 15.

In addition to established tumours, which would be identified as osteosarcomas by all experienced pathologists, we encountered a few lesions whose nature was not so clear. These took the form of localised foci of proliferating osteoblastic tissue, and were similar to those encountered in the injection experiment (Owen, Sissons and Vaughan, 1957) and then described as early sarcomas or " presarcomatous " lesions. In the present material these were found in the long bones (Fig. 16, 17) but were also encountered in relation to the bone surrounding mandibular and maxillary molar teeth (Fig. 18). It is necessary to be sure that some or all of these small lesions are not merely extensions from larger established tumours : while absolutely rigorous proof of this would demand complete serial sectioning of the involved bones, it can be stated that foci of this sort were encountered in sections in which there was no evidence of gross tumours. One interesting and apparently early lesion is the nodule of tumour occupying the marrow cavity of the mid-shaft of the tibia in rabbit 925 (Fig. 17). This consists of proliferating osteoblastic tissue, and appears to be continuous with a layer of newly formed bone that is present on the endosteal surface of the shaft in this situation.

An interesting histological feature of some of the established tumours was the type of regional variation in histological structure that is illustrated by the lesions shown in Fig. 5, 10, 11, 12, 13 and Fig. 6, 14, 15, 19. In Fig. 5 the lower end of the femur of rabbit 932 is occupied by a mass of cellular, invasive and obviously malignant tumour tissue : the shaft, in contrast, is occupied by a network of highly differentiated bone tissue (Fig. 11) which can be recognised as invasive only at its upper margin where tumour cells are infiltrating the spaces between fat cells of the marrow (Fig. 12). Do these contrasting areas represent independent tumours, or parts of the same lesions? If the latter, what is the relationship between them? A similar problem is posed by the existence, within the humerus of the same rabbit (932), of a number of different types of apparently neoplastic tissue (Fig. 6, 14, 15, 16, 19). The largest of these (Fig. 6) consists of a central mass of frankly malignant tumour tissue showing little new bone formation. Above this (Fig. 16), there is a patch of apparently separate osteoblastic tissue : this is interpreted as representing an independent neoplastic focus. But below the main lesion, and continuous with it, is a most unusual type of osteoblastic tissue (Fig. 14, 15), which, though cellular, can only be recognised as invasive at its lower margin (Fig. 19). Here, as with the tumour of the femur, the relationship between the two contrasting areas of tumour tissue is of interest.

3. *A study of the upper half of the tibia*

As in previous work on normal rabbits and rabbits given a single injection of $^{90}Sr$ the upper half of the tibia has been studied in much greater detail than any other bone. The distribution of the retained $^{90}Sr$ as seen on autoradiographs of thin sections and the radiation damage as seen on microradiographs and some histological sections is discussed here. The results of the measurement of radiation dose-rate and its relation to damage in different parts of the bone are described in detail elsewhere (Owen and Vaughan, 1959).

(i) *Distribution of $^{90}Sr$. Weanling rabbits.*—The results are best described with reference to Fig. 20 which shows 2 diagrams of the upper half of the tibia of an adult rabbit (i.e. aged 6 months plus). In Fig. 20*a* are shown in black the main regions of $^{90}Sr$ retention in a rabbit fed $^{90}Sr$ from the age of 5–8 weeks. In Fig. 20*b* is shown the pattern of bone growth after the age of 5–8 weeks. It will be seen that $^{90}Sr$ is taken up throughout the bone formed after $^{90}Sr$ feeding started.

There is a slight simplification here since some $^{90}Sr$ is also always taken up in remodelling Haversian systems in the bone which existed before feeding started For a detailed description of the pattern of growth of the upper half of the normal

FIG. 20.—Diagrams of longitudinal section through the middle of the posterior wall P and the lateral medial corner LM of the upper half of the tibia of a fully-grown rabbit, showing main regions of $^{90}Sr$ retention in relation to bone growth.

rabbit tibia, reference should be made to earlier work (Owen, Jowsey and Vaughan, 1955). The numbers at the side of the diagram refer to the approximate levels of sections, mentioned in the text, from which microradiographs and autoradiographs were made.

It was found, however, that in the animals which survived for 200 days or more, the most recently-formed bone contained less $^{90}Sr$ than that which was formed soon after $^{90}$Sr feeding started. This point is illustrated in Fig. 21, where the most recently-formed bone (an endosteal band, lower right, and a periosteal band, upper left) shows a lower content of $^{90}$Sr than the greater part of the bone formed during the course of the experiment. There was also some variation in $^{90}$Sr content in different Haversian systems associated with variation in their calcium content as seen in microradiographs. Tumours containing calcified tissue held some $^{90}$Sr but much less than adjacent bone.

*Old rabbits.*—In these two rabbits, which died 162 and 431 days after starting $^{90}$Sr feeding, there was a diffuse distribution of $^{90}$Sr throughout the bone but in extremely low concentration. Heavy patchy uptake was seen on the edges of bone trabeculae and on the endosteal surfaces in the epiphysis (Fig. 22). Throughout the shaft there were local concentrations on the endosteal surface (Fig. 23) and occasionally on the periosteal surface and in Haversian systems.

*Offspring of rabbits fed $^{90}$Sr.*—Autoradiographs were not made on bone sections from the rabbits suckled for five weeks. In the rabbits born dead the whole tibia contained $^{90}$Sr but this was in higher concentration in the bone in the middle third than it was in the cartilage at either end.

(ii) *Radiation damage. Weanling rabbits.*—Microradiographs of cross-sections of the upper half of the tibia of the rabbits killed or dying less than 31 days after feeding started showed in general no abnormality. The tibia of rabbits fed for over 200 days showed considerable evidence of radiation damage. In some, extensive and obvious tumour extended from the epiphysis into the mid-diaphysis ; in others tumour was present but not so extensive. There was abnormal bone, particularly on the endosteal surface of the medial and posterior walls. This abnormal bone appeared in the microradiographs of cross-sections to have large lacunae and an extremely irregular appearance of the bone lamellae, while the endosteal surface showed varying degrees of irregularity. It extended along the length of the bone studied and it was impossible to say at which level it had originated. Such bone can be seen in Fig. 24 in the posterior-medial corner at level 42. This figure also illustrates the extreme irregularity of the surface suggesting that there is possibly early osteosarcoma. Fig. 25 shows a cross-section at level 28 from the same bone, at which level there was great proliferation of abnormal tissue, which could be seen to fill the whole cavity when the section was cut. It was not all calcified and therefore does not show in the microradiograph. When stained, this section had an appearance like that seen in Fig. 12. There was highly differentiated tumour bone between the fat cells of the marrow. Fig. 26 shows a high power view of the endosteal surface in the medial-lateral corner at level 15, again from the same bone. Here are seen again the abnormal enlarged lacunae and an irregular surface. Histological preparations from such a site showed the lacunae to be empty and the bone to stain poorly, while the irregular surface was often associated with osteosarcomatous tissue showing varying degrees of bony differentiation.

Other changes took the form of scattered but patchy loss of osteocytes, which was sometimes, but not always, associated with damage to small blood vessels. The cell loss was often evident in the compact bone of the shafts of the long bones. In different animals and in different bones, the intensity of this change varied from minimal to obvious. The vascular changes took the form of occlusion of small blood vessels in Haversian spaces by fibrin clot, or were evident from an absence of any stainable vascular structures in the areas of bone damage.

The changes seen in microradiographs in bone formed on the periosteal surface were always less dramatic than those seen in microradiographs of endosteal bone. In no instance was tumour seen associated with the periosteal surface only, and in no instance was this surface irregular. The type of change in periosteal bone is shown in Fig. 27, a microradiograph from a section at level 25 (rabbit 932). It can be seen that buried some way below the present periosteal surface, there is a band with irregular arrangement of bone lamellae associated with enlarged lacunae. In the corresponding autoradiograph this area of bone is associated with the heaviest $^{90}$Sr concentration. In histological preparations at the same level the large lacunae contain no osteocytes, there may be some vascular damage and the bone stains poorly.

In the animal fed 25 $\mu$c daily which died 40 days later microradiographs showed areas of extremely low calcification particularly in lamellar bone and some abnormal periosteal bone. The metaphyseal trabeculae were devoid of active osteoblasts and there appeared to be an excessive amount of osteoclastic activity.

*Old rabbits.*—No radiation damage was noted in the microradiographs of the tibiae of these animals.

### III. *Teeth*

Observations on the uptake of $^{90}$Sr in the teeth, as seen in autoradiographs have been made on only a few animals. In a weanling rabbit killed 9 days after feeding started there was an even uptake of $^{90}$Sr in the roots of the teeth and in bone that had probably been laid down in that period. In rabbit 918, aged $3\frac{1}{2}$ years when feeding started and fed for 431 days, the mandible showed only slight patchy uptake characteristic of old bone but the teeth showed an even heavy uptake of $^{90}$Sr throughout their length (Fig. 28). This is in agreement with the fact that there is very little growth or remodelling in the bone of an animal of this age, whereas the teeth of the rabbit continue to grow throughout life. There was no histological damage in this case in either jaw bone or teeth, but the development of tumour in relation to the base of the molar teeth in other animals which were younger when feeding started has already been illustrated in Fig. 4 and the appearance of abnormal proliferating osteoblastic tissue eroding the dentine in Fig. 22.

### DISCUSSION

The present results indicate that, in weanling rabbits fed 8–11·8 $\mu$c $^{90}$Sr daily, multiple osteogenic sarcomas develop within 6–8 months in the rapidly growing ends of the long bones and the skull. Such tumours in the long bones are associated with abnormal endosteal bone surfaces extending from the epiphysis into the upper part of the diaphysis and with varying degrees of marrow aplasia. The widespread distribution of radiation dysplasia is associated with a widespread distribution of $^{90}$Sr in all bone formed after feeding started. This picture differs from that seen when $^{90}$Sr is given in a single injection, when there is only a localised region of intense radiation dysplasia in the same rapidly growing ends of the long bones which often become malignant, and show a high local concentration of $^{90}$Sr.

The relationship of radiation dose-rate and radiation damage in the two groups of animals is discussed in detail elsewhere (Owen and Vaughan, 1959) and has already been briefly summarised (Vaughan and Owen, 1959). Certain other points of interest are discussed below.

1. *The significance of the changes noted in the blood and in the haemopoietic organs*

The peripheral blood picture in rabbits fed for longer than 200 days was that associated with varying degrees of marrow aplasia ; in some cases the haemoglobin level was more affected, in others the white cells. In no instance was a true leukaemic blood picture seen, though a white cell count of 27,000 per c.mm. without any increase of abnormal white cells was once found in one rabbit ; in no instance was any blood loss noted. Though in some cases the terminal anaemia and leucopaenia were severe, a general gelatinous degeneration of the marrow was not found. In some cases patches of such gelatinous degeneration were present but the usual picture was of aplastic fatty marrow containing considerable areas of active haemopoiesis. The direct cause of death in all the younger rabbits at least was probably a haemopoietic one due to irradiation of the marrow by the $^{90}$Sr contained in the skeleton.

2. *The histogenesis of the bone tumours*

General factors concerning the production of bone tumours by radioactive materials and their histogenesis were considered in our previous account of the effects of injected $^{90}$Sr (Owen, Sissons and Vaughan, 1957). The tumours developing in the present experiment are very similar to those encountered previously. They are all osteosarcomas, and contain a variable amount of tumour bone. Their histological structure is generally comparable with that of human osteosarcomas, although some show an unusually high degree of structural differentiation.

Despite the clearly malignant local characteristics of the tumours, no metastases were observed in the present animals. In this connection it is of interest that only one of eight tumour-bearing animals in the injection experiment (Owen, Sissons and Vaughan, 1957) showed metastases, which were present in the liver. At first sight, this is in contrast with general experience with bone tumours produced with radioactive materials, whether in man (Martland, 1931) monkeys (Petrov *et al.*, 1952), rabbits (Schürch and Uehlinger, 1935 ; Ross, 1936 ; Hellner, 1937) or rats (Kuzma and Zander, 1957*a*) where metastases have frequently been observed. We believe, however, that in common with other radiation-induced bone tumours, those produced by $^{90}$Sr in rabbits are highly malignant, and that our animals, which were killed when bone tumours were recognised, or which died from other causes, would have developed metastases if they had survived longer.

With regard to the histogenesis of the tumours, the early lesions observed in our present material are in keeping with our suggestion (Owen, Sissons and Vaughan, 1957) that the tumours develop from the osteogenic connective tissue covering the surfaces of areas of abnormal bone. Proliferative " presarcomatous lesions " or " early sarcomas " of the type we reported in rabbits injected with $^{90}$Sr have been observed in the present material : they have also been reported by Litvinov (1957) in rats injected with $^{90}$Sr, and by Kuzma and Zander (1957*b*)

in rats injected with $^{45}$Ca or $^{90}$Sr. While their frequent occurrence in animals receiving high dosage of radioactive isotopes, and their histological similarity to more established tumours suggests very strongly that they represent a stage in the development of the later lesions, final decision regarding their exact nature and significance must still be deferred. Information on the dose-dependence of these proliferative lesions, and of other changes such as the peritrabecular fibrosis observed with alpha-emitting bone-seeking isotopes (Lisco, 1956; Jee, Arnold and Cochran, 1957) may clarify their relationship to tumour formation. If, for example, it is found that tumours develop at low dose levels which are not associated with these changes, it will be clear that they are not necessary precursors of tumour development. Studies with other animal species and with other bone-seeking isotopes will also be of importance in assessing the significance of these proliferative lesions.

Finally, the " composite " lesions noted in the present paper must be considered. It is possible that each of them represents two or more originally separate tumours which have later become confluent. Alternatively, and this is the interesting possibility, the areas of highly cellular pleomorphic tissue may represent areas of more rapid growth, with consequent loss of tissue differentiation, in a pre-existing more highly differentiated tumour.

3. *Quantitative retention of $^{90}$Sr in the skeleton*

It has already been pointed out that figures given in the tables for $^{90}$Sr retention in the whole skeleton are approximate, since they are calculated from measurements on only part of the skeleton. The values obtained for $^{90}$Sr in the tibia or femur, are all measured values and are therefore more reliable. They show that there is some variability among animals within the same group in $^{90}$Sr retention measured per g. bone or per g. Ca. This variability may occur because of differences in $^{90}$Sr intake due to secondary ingestion from coprophagy or because of variation in the daily calcium intake due to differences in appetite. The number of animals in the experiment is small so that it was not possible to determine whether the variability in $^{90}$Sr retention was related in any way to the age or weight of the animal when feeding started, or to the terminal calcium content of the bone.

As would be expected, the retention of $^{90}$Sr in the skeleton, expressed as a percentage of the amount fed, was greater in the rabbits that started feeding when 5–8 weeks old than in rabbits which started feeding when $3\frac{1}{2}$–4 years old. The retention of $^{90}$Sr in young rabbits fed for 9 days, 31 days and 224–280 days was, on average, 42, 46 and 10 per cent respectively of that ingested.

It should be noted in a comparison of the figures in Table I that rabbits fed for periods up to 9 days received 11·8 $\mu$c, the 31 day rabbits 8·0 $\mu$c and the long term rabbits on average 8·5 $\mu$c daily. Allowance being made for these differences in daily intake, it can be seen that the rate of increase of $^{90}$Sr per g. of Ca in the tibia is rapid up to 9 days, slightly slower from 9 till 31 days and much slower after 31 days. Under the present experimental conditions the quantity of $^{90}$Sr per gram of calcium, averaged as it is throughout the bone, depends in a complicated way on many factors. The two most important are: (*a*) the ratio of non-active (i.e. bone formed before feeding started) to radioactive bone (i.e. bone formed after feeding started), and (*b*) the ratio of the $^{90}$Sr to Ca in the blood. It is obvious that (*a*) has its greatest value at the start of $^{90}$Sr feeding and decreases thereafter, while (*b*) gradually decreases throughout the period of feeding. This decrease occurred because the amount of $^{90}Sr$ fed per day was approximately the same throughout the experiment while the Ca intake increased as the animal grew bigger. The rapid increase in the quantity, i.e. the amount of $^{90}Sr$ per gram of Ca, in the initial period of $^{90}Sr$ feeding is mainly due to the decrease in (*a*). Decrease in (*b*) would depress this quantity and would be particularly important in the latter stages of the experiment. Under ideal theoretical conditions, such as a constant $^{90}Sr/Ca$ ratio in the blood supply from the time of conception, the $^{90}Sr$ per gram of Ca in the bone would probably be constant with time.

The figures for $^{90}Sr$ content of the newborn rabbits and sucklings confirm previous observations, namely, that little $^{90}Sr$ passes from the mother's skeleton to the foetus but that considerable quantities of $^{90}Sr$ pass in the milk (Kidman, Tutt and Vaughan, 1951) probably coming from the $^{90}Sr$ of the mother's blood rather than from her bones. As would be expected ,$^{90}Sr$ retention was much less in the bones of rabbits four years old when feeding started than in younger animals.

Attention has been drawn elsewhere (Holgate, Mole and Vaughan, 1958; Holgate, 1959) to the heavy uptake of $^{90}Sr$ in the teeth in both injected and fed animals, and it has been suggested that the high incidence of tumours in the mandible and maxilla in either animals fed or injected may be due to radiation from $^{90}Sr$ concentrated in the teeth rather than in the bones themselves. It is to be noted that all five weanlings fed for more than 200 days had multiple tumours in the mandibles and maxillae associated with the roots of the molar or incisor teeth. There is also an even, high uptake in the teeth of an old rabbit fed for a long period, which is in striking contrast to the slight irregular uptake in jaw-bone. This suggests that in older animals the $^{90}Sr$ content of the teeth may present a greater hazard than the bone uptake.

4. *The pattern of distribution of $^{90}Sr$*

The present study, using autoradiographs of thin sections, has shown that in young rabbits fed $^{90}Sr$ the isotope is taken up in bone formed after the start of feeding (Fig. 24) and that within this bone the uptake is to some extent non-uniform. This unevenness is probably accounted for by two factors, (i) the decrease in the $^{90}Sr/Ca$ ratio of the diet in the course of feeding, mentioned above and (ii) the normal variation in calcium content of different parts of the bone. The variation in calcification in normal rabbit bone is not great and the size of the bone structures small so that in fact the uniformity of radiation dose-rate received by the bone is scarcely affected by this. The radiation dose-rate received by epiphyseal bone recently formed was found to be less than that received by bone formed soon after feeding started (Owen and Vaughan, 1959). This is due no doubt to the progressive decrease of the $^{90}Sr/Ca$ ratio in the diet.

The observations made here on the distribution of $^{90}Sr$ following daily feeding suggest that provided the $^{90}Sr/Ca$ content of the diet is constant the calcium of bone will be evenly labelled with $^{90}Sr$ in young rabbits.

In old rabbits the picture is different. In the bones of fully grown rabbits apposition occurs continuously but in a patchy way. When $^{90}Sr$ is fed there are rather more $^{90}Sr$-containing patches than when $^{90}Sr$ is given as a single injection, since the circulating blood level of $^{90}Sr$ is maintained, but the pattern of uptake is the same following both methods of administration.

SUMMARY

1. $^{90}Sr$ has been fed daily to 24 rabbits of different ages for varying periods at the rate of either 8–11·8 $\mu c$ or 25 $\mu c$ per day. Rabbits fed 8–11·8 $\mu c$ from the age of 5–8 weeks which survived for longer than 200 days developed multiple osteosarcomas at the growing ends of their long bones and in the bone surrounding maxillary and mandibular molar teeth. No skeletal lesions were seen in two rabbits over 3 years old when feeding started nor in sucklings born to rabbits fed $^{90}Sr$.

2. Varying degrees of anaemia, leucopaenia and thrombocytopaenia occurred in rabbits aged 5–8 weeks when $^{90}Sr$ feeding began but not in the older age group. The marrow showed variable degrees of aplasia in younger rabits, though areas of active haemopoiesis could always be found.

3. The bone tumours were osteosarcomas which appeared to arise from the whole endosteal surface of the more rapidly growing ends of the long bones, including the epiphysis. Two of the tumours had conspicuous vascular spaces.

4. Patchy bone necrosis (i.e. empty lacunae and deficient staining) was widespread, but such bone damage was not invariably associated with demonstrable occlusion of blood vessels. Neither fibrosis adjacent to bone surfaces nor gross failure of resorption was conspicuous.

5. On prolonged feeding of weanling rabbits with 8–11·8 $\mu c$ $^{90}Sr$ daily the rate of increase of $^{90}Sr$ per g. of calcium was at first rapid and then fell off with time. When the daily intake of $^{90}Sr$ was increased from 11·8 to 25 $\mu c$ daily the retention of $^{90}Sr$ per g. of bone or per g. of calcium increased in approximately the same ratio.

6. The distribution of $^{90}Sr$ in bone formed after feeding started was slightly uneven in young rabbits where the bones were growing in length, there being less in the more recently formed bone. The reasons for this are discussed. The distribution in the bones of old rabbits was patchy, as in old rabbits receiving $^{90}Sr$ by injection.

We are indebted to Professor Martin Rushton and Surgeon Captain W. Holgate, R.N., for comments on the pathological changes seen in the teeth, and to Mrs. S. Groves, Miss J. Gardner, Miss D. Gross and Miss F. Schofield, for technical help.

REFERENCES

Hellner, H.—(1937) *Arch. klin. Chir.*, **189**, 706.
Holgate, W.—(1959) *Brit. dent. J.*, **107**, in the press.
*Idem*, Mole, R. H. and Vaughan, J.—(1958) *Nature, Lond.*, **182**, 1294.
Jee, W. S. S., Arnold, J. S. and Cochran, T. H.—(1957) *Anat. Rec.*, **127**, 424.
Jowsey, J.—(1955) *J. sci. Instrum.*, **32**, 159.
Kidman, B., Rayner, B., Tutt, M. and Vaughan, J.—(1952) *J. Path. Bact.*, **64**, 453.
*Idem*, Tutt, M. L. and Vaughan, J.—(1950) *Ibid.*, **62**, 209.—(1951) *Ibid.*, **63**, 253.
Kuzma, J. F. and Zander, G.—(1957*a*) *Arch. Path.*, **63**, 198.—(1957*b*) *Amer. J. Path.*, **33**, 607.
Lamerton, L. F. and Harriss, E. B.—(1954) *J. photogr. Sci.*, **2**, 135.
Lisco, H.—(1956) *In* Ciba Foundation Symposium on Bone Structure and Metabolism, London, p. 272.
Litvinov, N. N.—(1957) *Arkh. Pat.*, **19**, No. 1, p. 26.

MARTLAND, H. S.—(1931) *Amer. J. Cancer*, **15**, 2435.
OWEN, M., JOWSEY, J. AND VAUGHAN, J.—(1955) *J. Bone Jt. Surg.*, **37 B**, 324.
*Idem*, SISSONS, H. A. AND VAUGHAN, J.—(1957) *Brit. J. Cancer*, **11**, 229.
*Idem*, AND VAUGHAN, J.—(1959) *Ibid.*, **13**, 424.
PETROV, N. N., KROTKINA, N. A., VADOVA, A. V. AND POSTNIKOVA, Z. A.—(1952) 'Dynamics of the Origin and Development of Malignant Growths in Experiments on Monkeys.' Moscow (Press of the U.S.S.R., Academy of Medical Sciences).
ROSS, J. M.—(1936) *J. Path. Bact.*, **43**, 267.
SCHÜRCH, O., AND UELINGER, E.—(1935) *Arch. klin. Chir.*, **183**, 704.
SHELDON-PETERS, J., AND VAUGHAN, J.—(1956) *Brit. J. exp. Path.*, **37**, 553.
SINCLAIR, W. K., ABBATT, J. D., FARRAN, H. E. A., HARRISS, E. B., AND LAMERTON, L. F.—(1956) *Brit. J. Radiol.*, **29**, 36.
VAUGHAN, J. AND OWEN, M.—(1959) *Lab. Invest.*, **8**, 181.